# From early development to maturity: a phenotypic analysis of the Townes sickle cell disease mice

**DOI:** 10.1242/bio.061828

**Published:** 2025-02-06

**Authors:** Ariadna Carol Illa, Henning Hvid, Torben Elm, Christa Andsbjerg Frederiksen, Lonnie Frimodt Bangshof, Dennis Funch Danielsen, Søren Skov, Carsten Dan Ley

**Affiliations:** ^1^ Rare Disease Research, Global Drug Discovery, Novo Nordisk A/S, 2760 Måløv, Denmark; ^2^ Department of Veterinary and Animal Sciences, Faculty of Health and Medical Sciences, University of Copenhagen, 1870 Frederiksberg C, Denmark; ^3^ Global Discovery & Development Sciences, Global Drug Discovery, Novo Nordisk A/S, 2760 Måløv, Denmark

**Keywords:** Sickle cell disease, Regenerative anaemia, Mouse models, Preclinical research, Histology, Artificial intelligence

## Abstract

Well-characterised mouse models of disease may provide valuable insights into pathophysiology. This study characterises the Townes mouse model of sickle cell disease (SCD) and establishes a time window in which the disease is present but does not progress significantly in terms of severity. We examined Townes mice with the HbAA, HbAS, and HbSS genotypes from young (4 weeks) to mature (5 months) stages of life to assess the disease state at different ages and any progression. We conducted blood tests, histological organ damage evaluations, and metabolic assessments to identify a suitable time frame for study based on welfare considerations. Townes HbSS mice displayed key SCD features such as anaemia, haemolysis, thromboinflammation and organ pathology. Notably, these manifestations remained relatively stable over the study period, indicating a stable phase suitable for conducting intervention studies. Mice with HbAS and HbAA genotypes served as comparative controls, showing minimal to no pathology throughout. These findings are valuable for future research on SCD and may ultimately lead to the development of more effective treatments for this debilitating disease.

## Teaser

**Summary:** This longitudinal characterisation provides insights into the pathophysiology of sickle cell disease in the Townes mouse model, which is useful for guiding design and interpretation of preclinical studies and is of potential relevance in understanding disease progression.

## INTRODUCTION

Sickle cell disease (SCD) is a genetic disorder affecting an estimated 17 million people worldwide, mainly in sub-Saharan Africa. Each year, over 500,000 individuals are born with the disease (GBD 2021 Sickle Cell Disease Collaborators, 2023; Piel et al., 2013; Sickle cell disease, CDC). SCD is caused by a mutation in the gene encoding the β subunit of haemoglobin (*HBB*). This mutation is characterized by an A to T transversion in the sixth codon, resulting in the formation of the sickle allele Hβ^s^ (Ingram, 1956, 1957). Under deoxygenation, the sickle haemoglobin tetramers (HbS; α_2_β^S^_2_) polymerise, leading to erythrocyte damage and deformation into the sickle shape. These sickled cells are prone to haemolysis and occlusion of small capillaries, further exacerbating the disease (Steinberg, 2008; Noguchi and Schechter, 1981; Hofrichter et al., 1974). The combination of other pathophysiological mechanisms, such as vaso-occlusion and immune activation, leads to remarkable disease complexity. SCD can lead to a range of serious complications, including stroke, kidney failure, lung and liver disorders, infection, anaemia, painful crises, and other chronic conditions that can cause organ damage (Rees et al., 2010; Kato et al., 2018).

SCD presents shortly after birth, at around 3 months of age, after the haemoglobin switch from fetal (HbF) to adult (HbS) occurs (Sankaran and Orkin, 2013). Despite therapeutic advancements, SCD patients continue to experience significant morbidity and average life expectancy is 52.6 years, with males slightly lower than females (Jiao et al., 2023). This highlights the need for further advancements in the management of SCD to improve outcomes and quality of life for those affected by the disease.

Animal models can be pivotal in bridging the gap between basic research and clinical studies, enabling a deeper understanding of the complex pathophysiology of SCD and evaluation of potential therapies. The first described SCD model was the SAD mouse, which contains a β^6^ Val substitution of the β^S^ chain, as well as S-Antilles-D Punjab mutations. Although this model displays certain disease characteristics, such as glomerulopathy, mice are not anaemic in adulthood and the model is considered mild (De Paepe and Trudel, 1994; Trudel et al., 1991).

Two other commonly used mouse models of SCD are the Berkeley (BERK) and Townes models, which express human globin genes instead of the mouse paralogs (Pászty et al., 1997; Wu et al., 2006). The Berkeley mouse harbours three DNA transgenes expressing the human α-, β^s^- and γ-globin genes. However, recent evidence suggests the insertion of these genes may be semi-random and fragmented, resulting in variable globin expression, which challenges the model's effectiveness for certain therapeutic studies, such as HbF induction and gene editing (Woodard et al., 2022). Despite some differences from human SCD, the Berkeley mice display severe manifestations of SCD including haemolysis, vascular congestion, ischemic changes, and multiorgan dysfunction. Notably, the lack of protection by HbF at birth leads to high neonatal mortality (Beuzard, 2008).

The Townes mouse was created using homologous recombination by replacing the mouse α-globin genes with human α-globin (*HBA1*) and the mouse β-like globin genes with tandemly linked genomic segments of human γ-globin (*HBG1*) and sickle β-globin (*HBB^s^*), as well as some known proximal regulatory elements, but key DNA-regulatory elements are missing (Wu et al., 2006; Woodard et al., 2022). Unlike the Berkeley model, the Townes mice recapitulate the fetal to adult Hb conversion that happens in humans after birth, therefore being protected by HbF during embryonic development (Edoh et al., 2006; Sankaran and Orkin, 2013). This model has been supportive in elucidating the pathophysiology of SCD, with homozygous HbS Townes mice (hα/hα::βS/βS) displaying severe anaemia, red blood cell sickling, altered nociception, and multi-organ dysfunction, while heterozygous mice (hα/hα::βA/βS) mirror the mostly asymptomatic human sickle cell trait, and homozygous normal mice (hα/hα::βA/βA) serve as phenotypically normal controls (Nguyen et al., 2014; Lei et al., 2016; Alvarez-Argote et al., 2023; Zappia et al., 2017; Trucas et al., 2023).

In spite of these findings, quantitative understanding of disease progression and the impact of ageing in these models is limited. Our study aims to fill this gap by characterizing the state of the disease in the Townes mouse model from a young age (4 weeks) to maturity (22 weeks), in order to determine the age range where studies of these mice would be feasible without unnecessarily compromising animal welfare. Mice are considered mature adults between 3 and 6 months of age, as they are not yet affected by senescence, and become sexually mature by 35 days (Flurkey et al., 2007). To achieve our goal, we studied Townes mice with the sickling phenotype (homozygous for hα/hα::β^S^/β^S^, here HbSS), Townes carriers (heterozygous hα/hα::β^A^/β^S^, here HbAS) and Townes wild-type mice (homozygous for hα/hα::β^A^/β^A^, here HbAA) at each month from 1 to 5 months of age. We assessed their phenotypes by evaluating haematological and metabolic markers.

Furthermore, as there is limited histological quantitative data showing a measurable state of the disease, we also developed an image analysis algorithm based on artificial intelligence (AI), trained to classify and detect different tissue types automatically in liver Haematoxylin and Eosin (H&E) slides, providing a quantitative way to assess tissue damage.

Data from two distinct studies are presented in this manuscript: the first study followed Townes mice from 4 weeks of age until 12-13 weeks of age with a staggered take-down of mice every week, to assess changes in disease state in the young/juvenile mice. The second study followed Townes mice from 8 weeks of age until 22 weeks of age to assess changes in disease state as the mice developed into full maturity. We present the results as a meta-analysis of both studies.

## RESULTS

### Consistent anaemia and splenomegaly in sickling mice, despite normal growth

To investigate the progression and effects of sickle cell disease, we measured body and spleen weights, conducted spleen histology, and analysed haematological parameters in Townes mice. Weights and platelet counts are reported separately for males and females due to observed significant differences between sexes; unless statistically significant, males and females are plotted and reported together in this and all following figures. All *P*-values from the studied comparisons can be found in Table S1.

**Fig. 1.**
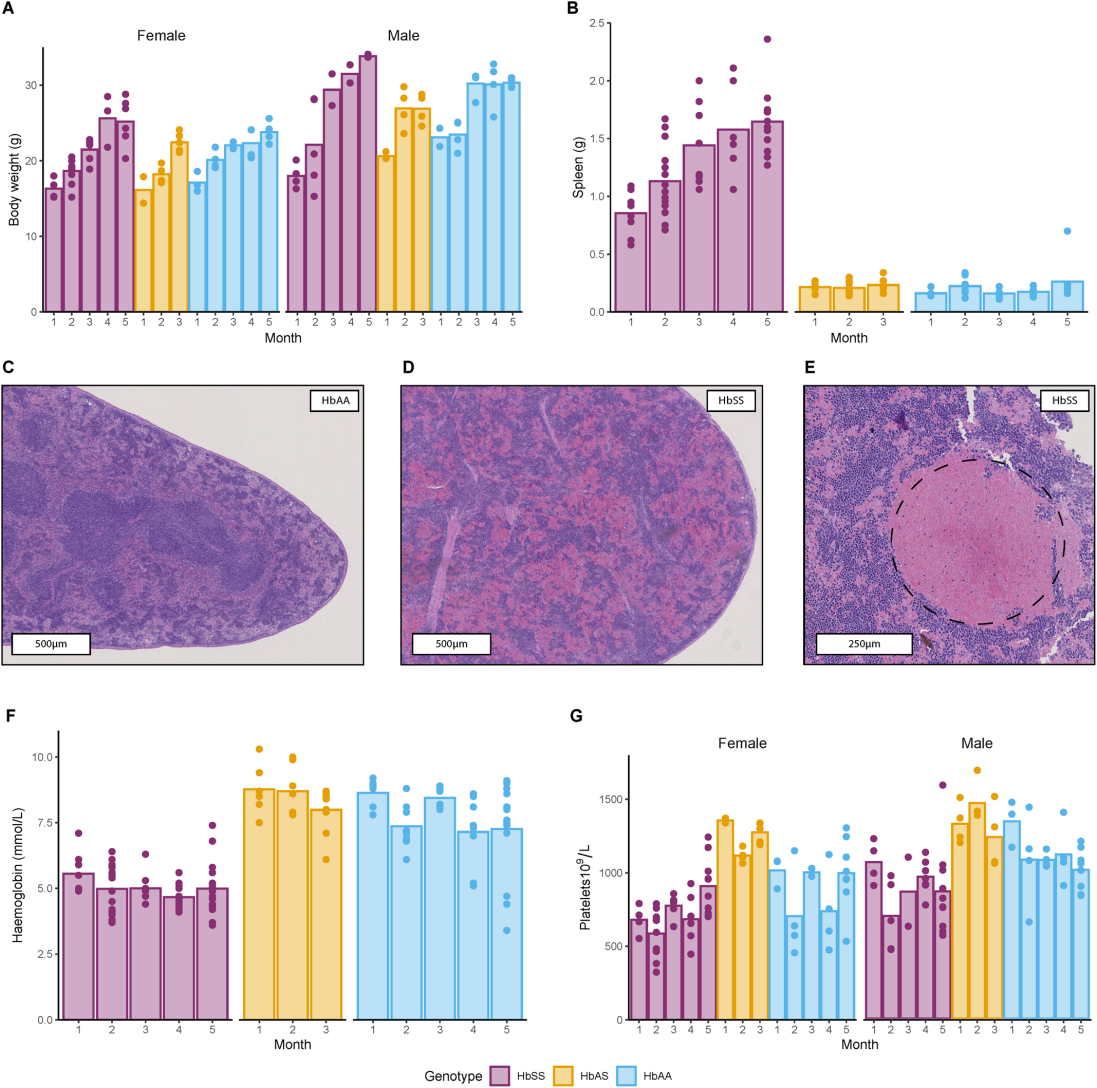
**Clinical presentation and anaemia status by age and genotype.** Bars represent the mean and each dot represents an individual animal. (A) Body weight (g) at termination. (B) Spleen weight (g). (C,D) H&E staining. Representative spleen images from HbAA and HbSS mice, respectively. Note the red pulp expansion in D. (E) Close-up of H&E staining in HbSS. Enlarged view showcasing the presence of a thrombus (dashed circle). (F) Haemoglobin levels (mmol/l) and (G) platelet levels (10^9^/l). Comparisons between genotypes, sex, and time for each parameter were made with a heteroscedastic ANOVA with adjustments for multiple tests (Tukey method) for *P*-values, refer to Table S1. Exact sample size (individual animals):

No differences in body weight (Fig. 1A) were observed between genotypes; however, compared to month 1, significant increases were observed in months 3, 4 and 5 for all genotypes, reflecting growth as expected for mice of this age. Notably, despite the disease manifestations, HbSS mice gained weight at a rate comparable to that of the wild-type mice.

**Table:** 

Parameter	HbSS (*n*)					HbAS (*n*)			HbAA (*n*)				
Month	1	2	3	4	5	1	2	3	1	2	3	4	5
Body weight	8	15	8	6	12	5	8	9	5	8	6	8	8
Spleen	8	15	8	6	12	6	8	9	6	8	6	8	8
Hb and PLT	8	15	8	14	20	6	8	9	6	8	6	8	16

Despite the absence of genotype-based differences in body weight, spleen weights (Fig. 1B) were significantly elevated in the sickling (HbSS) mice compared to both carrier (HbAS) and wild-type (HbAA) mice (*P*<0.0001 for both comparisons), as expected, and significantly increased over time. Interestingly, the spleen-to-body weight ratio (Fig. S1A), remained constant in all genotypes.

Histological examination of the spleen showed expansion of the red pulp with accumulation of sickle cells and increased extramedullary haematopoiesis, which was found in homozygote animals of all ages (Fig. 1D). Lymphoid follicles were completely absent in most HbSS animals, although a few follicles were observed in the remaining HbSS animals. Furthermore, thrombus formation (Fig. 1E) and mild necrosis were present in homozygotes. None of these findings were observed in HbAA mice (Fig. 1C).

A characteristic phenotype of SCD is anaemia and haemolysis, which can be observed in the HbSS genotype (Wu et al., 2006; Macharia et al., 2018). Total haemoglobin concentrations (Fig. 1F) were significantly lower in HbSS mice (5.0±0.8 mmol/l, mean±s.d.) compared to HbAS (8.4±0.9 mmol/l, *P*<0.0001) and HbAA mice (7.6±1.3 mmol/l, *P*<0.0001). Haemoglobin levels in HbSS and HbAS mice remained stable over time, whereas a slight yet statistically significant decrease was observed in HbAA mice at months 4 and 5 compared to month 1 (*P*=0.0463 and *P*=0.0323, respectively; Fig. 1F). Both HbAA and HbAS levels were within the 7.88-9.37 mmol/l established reference range for C57Bl/6 mice according to the literature, while HbSS haemoglobin levels were lower (Stahl et al., 2018; Mazzaccara et al., 2008; Santos et al., 2016).

Platelet counts were significantly different between all genotypes (*P*<0.0001), but no change over time was observed. Mean values for HbSS mice were 800.2±229.9×10^9^/l, for HbAS mice were at 1289.7±159.8×10^9^/l and for HbAA were 1001.3±249.1×10^9^/l. Also, there was a significant difference in platelet counts between male and female mice (Fig. 1G, *P*<0.0001), independently of the genotype. This is also reported in literature reference values for various mouse strains (Mazzaccara et al., 2008; Stahl et al., 2018).

### Red blood cell parameters are stable with increasing age and reflect regenerative anaemia in sickling mice

**Fig. 2.**
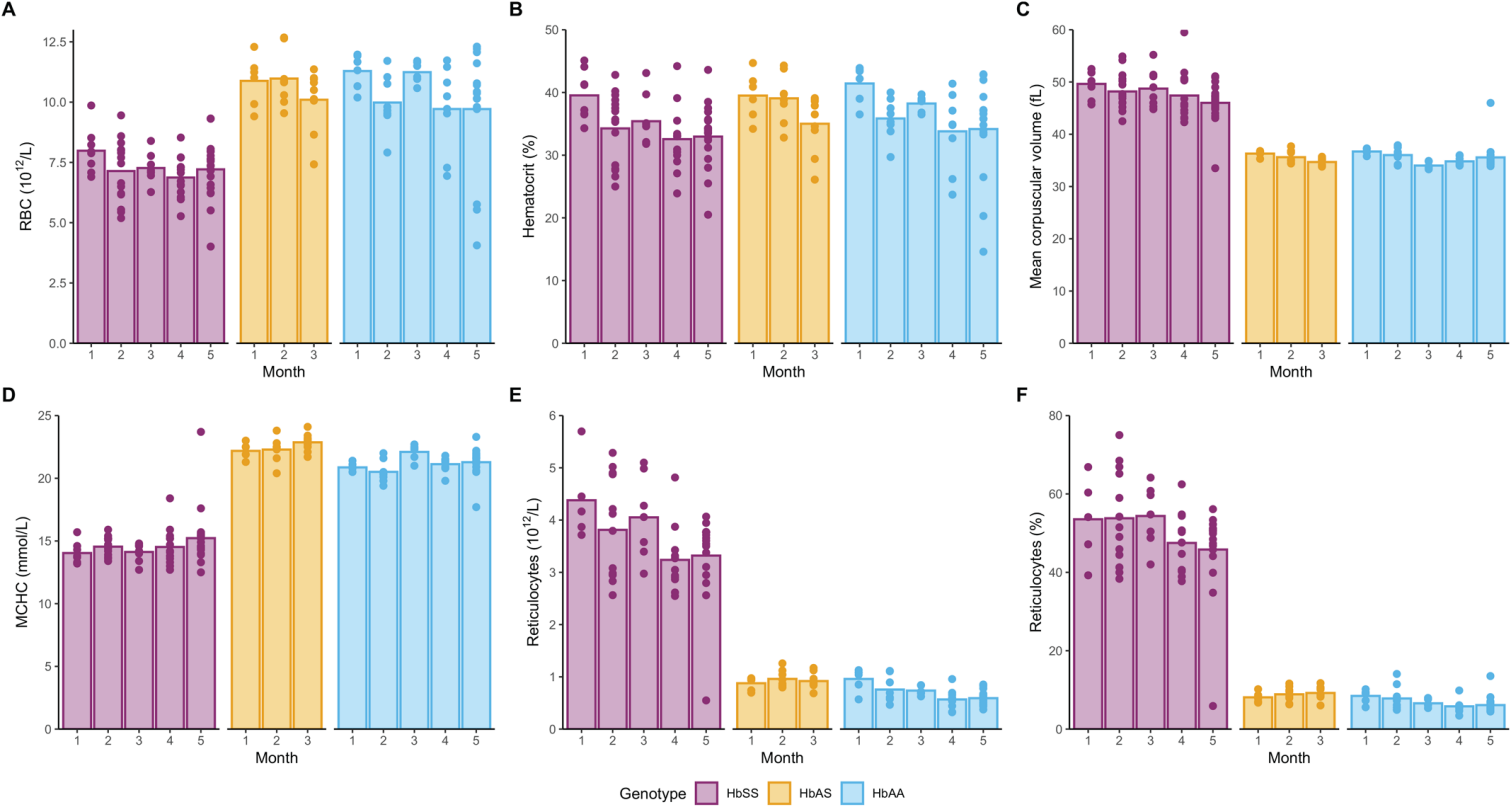
**Haematological parameters by age and genotype.** Bars represent the mean and each dot represents an individual animal. (A) RBC counts (10^12^/l). (B) Haematocrit (%). (C) Mean corpuscular volume (fL). (D) Mean corpuscular haemoglobin concentration (mmol/l). (E) Absolute reticulocyte count (10^12^/l) and (F) proportion of reticulocytes as of total RBC (%). Comparisons between genotypes, sex, and time for each parameter were made with a heteroscedastic ANOVA with adjustments for multiple tests (Tukey method) for associated *P*-values, refer to Table S2. Exact sample size:

Red blood cell (RBC) counts (Fig. 2A) were significantly lower in the sickling genotype (7.2±1.1×10^12^/l) compared to the other two genotypes (10.6±1.2×10^12^/l for HbAS and 10.2±1.8×10^12^/l for HbAA, *P*<0.0001 for both comparisons). This is consistent with the known anaemic phenotype and regenerative anaemia in these mice (Wu et al., 2006; Nguyen et al., 2014). RBC counts for HbAS and HbAA are within the range of literature values for C57Bl/6 mice (mean around 8.50-9.95×10^12^/l), hence they can be considered normal (Stahl et al., 2018; Mazzaccara et al., 2008; Santos et al., 2016). There were no significant change of RBC counts over time in any of the genotypes, indicating no change of the anaemic condition of the sickling mice. All *P*-values from Fig. 2 can be found in Table S2.

**Table:** 

Parameter	HbSS (*n*)					HbAS (*n*)			HbAA (*n*)				
Month	1	2	3	4	5	1	2	3	1	2	3	4	5
RBC, HCT, MCV, MCHC	8	15	8	14	20	6	8	9	6	8	6	8	16
Reticulocytes and % of reticulocytes	5	13	7	11	17	6	8	9	6	8	6	8	15

A small but statistically significant decrease in haematocrit (Fig. 2B) was observed for HbSS and HbAA between month 1 and month 5 (*P*=0.0220 and *P*=0.0294, respectively), as well as between month 1 and 4 for HbSS (*P*=0.0215). Overall, there were no differences between genotypes.

The observed mean corpuscular volume (MCV) (Fig. 2C) was higher in HbSS mice (47.6±3.9 fl) compared to the other genotypes (35.4±1.1 for HbAS and 35.5±2.0 for HbAA, *P*<0.0001). Literature values for C57Bl/6 mice and sickling Townes mice show a range of MCV values similar to HbSS mice in our study (46.7-55.3 fl) (Santos et al., 2016; Stahl et al., 2018; Alvarez-Argote et al., 2023; Lebensburger et al., 2010). A modest, but statistically significant change was observed between month 1 and 5 for HbSS (*P*=0.03).

Mean corpuscular haemoglobin concentration (MCHC) (Fig. 2D) was lower in HbSS mice (14.6±1.5 mmol/l) compared to HbAS (22.5±0.8) and HbAA (21.2±1.0), and all genotypes were significantly different from one another (*P*<0.001 for each of the comparisons), consistently across all age groups. A similar picture is observed for mean corpuscular haemoglobin (MCH) (Fig. S1B).

Consistent with regenerative anaemia, reticulocyte counts (Fig. 2E) were highly elevated in HbSS mice compared to the other genotypes (*P*<0.0001), accounting for 50% of the total red blood cells in HbSS mice (Fig. 2F). Significant decreases were detected in HbSS mice at both months 4 and 5 when compared to month 1 (*P*=0.0031 and *P*=0.0038, respectively). Despite the statistical significance of these reductions, reticulocyte counts remained considerably elevated. Interestingly, there was no significant change in the percentage of reticulocytes over time (Fig. 2F).

High fluorescent reticulocytes (HFR) account for the most immature fraction of reticulocytes. The percentage of HFR (Fig. S1C) was elevated for HbSS mice compared to the other two genotypes (*P*<0.0001). No significant differences over time were observed for HbSS and HbAA, but for HbAS at month 2 was found lower compared to month 1 (*P*=0.0012). Low fluorescent reticulocytes (LFR), representing more mature reticulocytes, showed the opposite pattern across genotypes, lower for HbSS compared to the other two (Fig. S1D). Furthermore, we investigated the thrombin generation potential of these mice (Fig. S2). HbSS mice exhibited significantly elevated peak thrombin levels and endogenous thrombin potential compared to wild-type Townes mice (Fig. S2A,B). However, the lag time and time to peak thrombin (data not shown) did not differ between the genotypes, indicating that while the overall thrombin generation capacity is increased in HbSS mice, the initial rate of thrombin generation remains unchanged.

### White blood cell populations are consistently elevated in sickling Townes mice

**Fig. 3.**
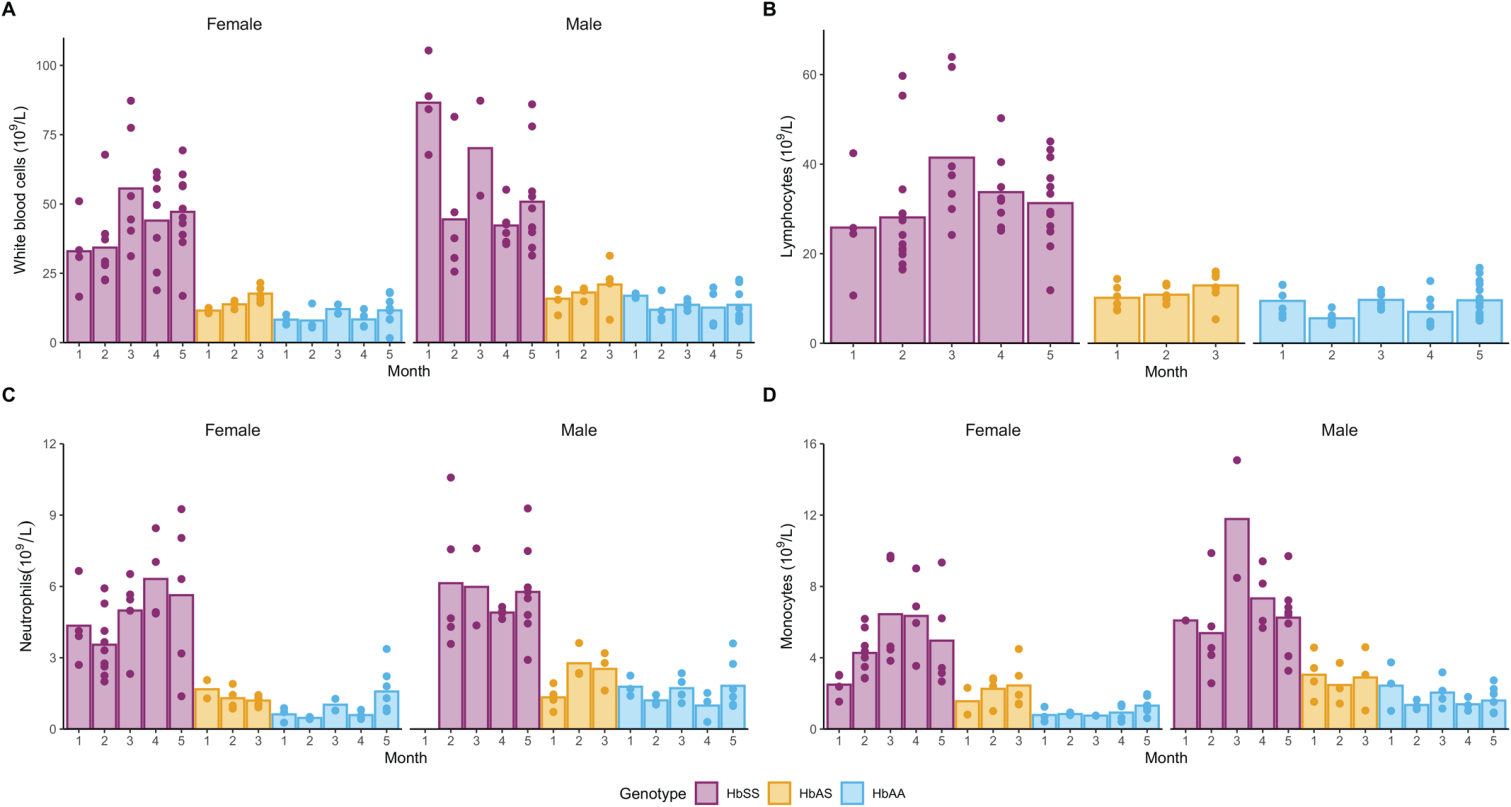
**WBC profiles by age and genotype.** Bars represent the mean, and each dot represents an individual animal. (A) WBC counts (10^9^/l). (B) Lymphocytes (10^9^/l). (C) Neutrophils (10^9^/l) and (D) monocytes (10^9^/L). Comparisons between genotypes, sex, and time for each parameter were made with a heteroscedastic ANOVA with adjustments for multiple tests (Tukey method), *P*-values are provided in Table S3. Exact sample size:

Overall, HbSS animals presented with elevated white blood cell (WBC) counts, including individual cellular subtypes such as lymphocytes, neutrophils and monocytes, compared to the other genotypes (Fig. 3, for *P*-values refer to Table S3). Male mice had higher WBC (*P*=0.004), neutrophil (*P*=0.0057) and monocyte (*P*=0.0007) counts than female mice (Fig. 3A,C,D). In HbSS mice, lymphocytes were significantly elevated, and significant increases were observed between months 1 and 3 (*P*=0.0269). Interestingly, although lymphocytes are the dominant cell type in mouse WBC population, lymphocyte counts (Fig. 3B) did not significantly differ between sexes (*P*=0.401), while all the other leukocyte counts did.

**Table:** 

Parameter	HbSS (*n*)					HbAS (*n*)			HbAA (*n*)				
Month	1	2	3	4	5	1	2	3	1	2	3	4	5
WBC	8	15	8	14	20	6	8	9	6	8	6	8	16
Lymphocytes, neutrophils and monocytes	4	14	7	8	13	6	7	8	6	6	6	7	14

Elevated levels of cytokines such as TNF-α, IL-1β, IL-6, and IL-8 have been reported in patients with SCD. Therefore, we investigated plasma cytokine levels in a subset of animals (Fig. S3).

Our analysis did not reveal any significant differences in IL-5, KC/GRO, IL-1β and IFN-γ between genotypes (Fig. S3B,E,F,H), consistent with published data (Alvarez-Argote et al., 2023). However, for TNF-α (Fig. S3A), despite the small dataset, we found significant elevations in HbSS mice compared to HbAS at month 3 (*P*=0.032) and for both month 2 and month 3 compared to month 1 for HbSS (*P*=0.031 and *P*=0.008, respectively).

Our data also indicated that IL-6 measurements (Fig. S3C) were significantly elevated in HbSS mice compared to HbAS (*P*=0.001), although the values remained within the normal range for mice [(MSD), 2013]. Finally, IL-10 (Fig. S3D) levels were significantly elevated in HbSS mice compared to HbAA (*P*=0.038) and IL-2 (Fig. S3G) levels were significantly higher in HbSS than HbAS (*P*=0.04) despite some mice being below the lower limit of quantification (LLOQ). Although we also measured IL-4 and IL-12p70, all mice fell below the LLOQ.

### Hepatic pathology and endothelial activation markers are affected in sickling mice

**Fig. 4.**
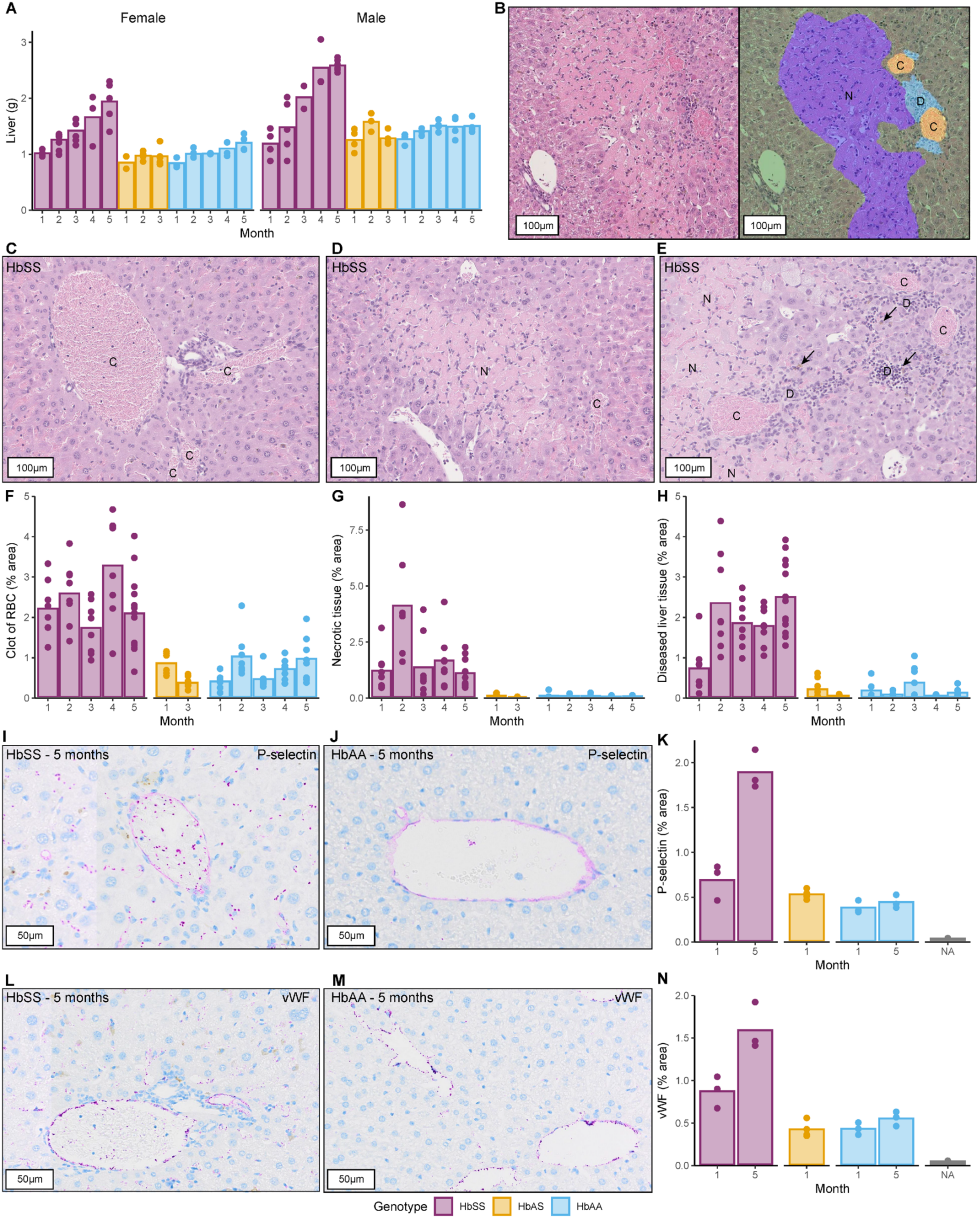
**Liver weight and histopathological characterization by age and genotype.** Mean values are depicted by bars, with individual animals represented by dots. (A) Liver weight (g) for female and male mice across different ages. (B-E) Liver sections stained with H&E showing: (B) algorithm-based classification of the tissue (left: unclassified, right: classified), (C) RBC clots (20x), (D) necrotic tissue (20x), and (E) diseased liver tissue with iron deposits indicated by arrows (20x, C for RBC clot, N for necrotic tissue, D for diseased liver tissue). (F-H) Quantitative analysis of histological features classified by the algorithm: (F) percentage of area identified as RBC clots, (G) percentage of area identified as necrotic tissue, and (H) percentage of area identified as diseased liver tissue. (I-K) Immunohistochemical detection of P-selectin in liver sections: (I,J) representative image from a 5-month-old mouse (20x), HbSS and HbAA, respectively, and (K) quantification of P-selectin-positive areas. (L-N) Immunohistochemical detection of von Willebrand factor (vWF) in liver sections: (L,M) representative image from a 5-month-old mouse (20x), HbSS and HbAA, respectively, and (N) quantification of vWF-positive areas. Comparisons between genotypes, sex, and time for each parameter were made with a heteroscedastic ANOVA with adjustments for multiple tests (Tukey method) for *P*-values, refer to Table S4. Exact sample size:

Liver weight was comparable between genotypes at months 1 and 2 (Fig. 4A), but from month 3 and onwards was significantly elevated for HbSS mice (*P*=0.0091 HbSS versus HbAS for month 3; *P*<0.0001 HbSS versus HbAA for months 4 and 5). Male livers were generally heavier than female livers (*P*<0.0001). The liver-to-body weight ratio (Fig. S1E) illustrates a similar pattern, with a significant increase observed in HbSS mice between months 1 and 5 (*P*=0.0002) and higher liver-to-body weight ratio in male compared to female mice (*P*=0.006). Macroscopically, livers of HbSS mice had a nutmeg-like appearance, indicative of congested vessels due to impaired blood flow.

**Table:** 

Parameter	HbSS (*n*)					HbAS (*n*)			HbAA (*n*)				
Month	1	2	3	4	5	1	2	3	1	2	3	4	5
Liver	8	15	8	6	12	6	8	9	6	8	6	8	8
% RBC clot, % necrotic tissue and % diseased liver tissue	8	8	8	8	13	8	NA	8	6	8	8	8	8
% of vWF and % of P-selectin	3	NA	NA	NA	3	4	NA	NA	3	NA	NA	NA	3

The AI-based algorithm trained to classify the liver tissue in digital images of the histological sections, was applied to H&E-stained liver sections from Townes mice of different ages and genotypes. The outputs of the algorithm were plotted and quantitatively assessed based on the proportion of the area corresponding to four distinct histological categories (Fig. 4B): normal liver tissue (green), RBC clots (orange), necrotic tissue (purple), and diseased liver tissue (light blue). Normal tissue prevalence was markedly reduced in HbSS mice, averaging 94±2.5%, in contrast to HbAS and HbAA mice, which exhibited averages of 99.3±0.5% and 99.1±0.5%, respectively (*P*<0.0001 for both comparisons). Fig. 4B provides a visual comparison, showcasing a representative section with the original H&E staining on the left and the algorithm-classified image on the right. Fig. 4C-E include sample images illustrating each histological category as identified by the algorithm.

Fig. 4F shows quantification of the area occupied by RBC clots in the tissue scans. As expected, a higher RBC clot area was observed in HbSS mice compared to the other two genotypes (*P*<0.0001), with the presence of sickled red blood cells. No significant differences between timepoints were observed within HbAA and HbAS. For HbSS, statistically significant changes in both directions were observed between month 1 and 4, 3 and 4, and 4 and 5 (*P*=0.0151, *P*=0.0001 and *P*=0.0013).

No necrotic tissue was observed in HbAA and HbAS (*P*<0.0001, for both comparisons against HbSS), as expected, since chronic tissue damage should not occur (Fig. 4G). For the HbSS mice, an elevation for month 2 was observed, statistically significant compared to the other months (*P*<0.0001 month 2 versus 1, 3, 4 and 5); however, since this was based on relatively few observations and was not sustained at later timepoints, we are uncertain how to interpret this.

Fig. 4H shows the quantification of diseased liver tissue, which includes presence of inflammatory cells, bile duct proliferation, and tissue irregularities that are not seen in healthy liver. Overall, HbSS mice have a greater area of diseased liver tissue than HbAS and HbAA (Fig. 4H, *P*<0.0001 for both comparisons), and for HbSS month 1 is significantly lower than all the other months (*P*-values in Table S4).

P-selectin is a cell adhesion molecule present on activated endothelial cells and platelets, and is involved in the pathophysiology of vaso-occlusive crises (VOCs) in SCD. Adhesion of whole blood and leukocytes to P-selectin is significantly increased during VOC events compared to stable periods (Pittman et al., 2021). We stained liver tissue from 1- and 5-month-old animals for P-selectin by immunohistochemistry (IHC) and quantified the stained area (Fig. 4I,J, representative images). Normally, P-selectin is expressed in the vascular endothelium in all genotypes, but in sickling mice it was also observed more widely dispersed within the tissue, in the sinusoidal space (Fig. 4I). For HbSS animals, P-selectin levels were elevated in both month 1 and particularly month 5 (compared to HbAA, *P*=0.046 and *P*<0.0001, Fig. 4K), and the level was significantly higher in 5-month-old HbSS animals compared to 1-month-old animals of the same genotype (*P*<0.0001, Fig. 4K).

Von Willebrand Factor (vWF) is involved in coagulation, and in SCD, vWF levels are reported to be elevated due to ongoing haemolysis and endothelial dysfunction; increased vWF levels have also recently been found associated with VOCs and acute chest syndrome (Sins et al., 2017; Shi et al., 2022; Vital and Lam, 2023). vWF levels in the liver were studied by IHC (Fig. 4L,M, representative images). As observed for P-selectin, in HbSS the levels at month 5 were increased compared to month 1 (*P*<0.0001). vWF was mainly detected in the vessel lumen but in HbSS mice it was also observed in the sinusoidal space (Fig. 4L), similarly to P-selectin. Overall, vWF levels were significantly increased in HbSS mice compared to HbAA and HbAS at both investigated timepoints (*P*=0.001 versus HbAA and *P*=0.0045 versus HbAS, Fig. 4N).

### Liver metabolic parameters are elevated in sickling mice

**Fig. 5.**
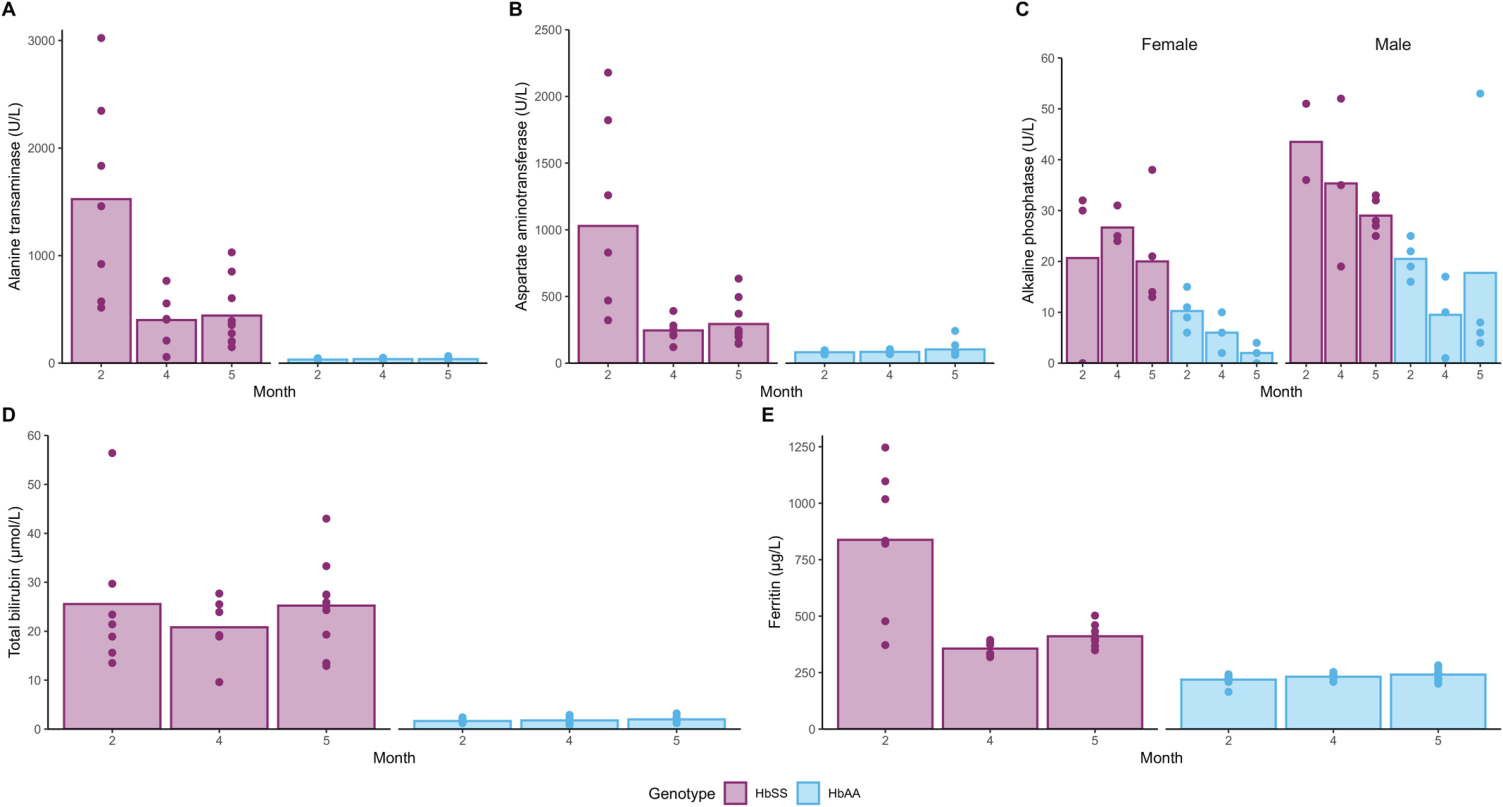
**Hepatic parameters by age and genotype.** Each bar represents the mean value and dots indicate the individual animals. Plasma levels of (A) alanine transaminase (ALT, U/L), (B) aspartate aminotransferase (AST, U/L), (C) alkaline phosphatase (ALP, U/L) separated by male and female, (D) total bilirubin (µmol/l) and (E) ferritin (µg/l). Comparisons between genotypes, sex, and time for each parameter were made with a heteroscedastic ANOVA with adjustments for multiple tests (Tukey method), for *P*-values, see Table S5. Exact sample size:

Alanine transaminase (ALT) and aspartate aminotransferase (AST) are liver disease markers, which we evaluated at 2, 4 and 5 months for HbSS and HbAA mice. Overall, levels were increased in HbSS mice (Fig. 5A,B) compared to HbAA mice (*P*<0.0001 for ALT and *P*=0.0002 for AST). Interestingly, for HbSS mice both ALT and AST levels decreased significantly at later timepoints (*P*<0.0001 compared to the 2-month-timepoint), although they remained well above the level of HbAA mice. All relevant *P*-values can be found in Table S5.

**Table:** 

Parameter	HbSS (*n*)			HbAA (*n*)		
Month	2	4	5	2	4	5
ALT, AST, ALP, total bilirubin and ferritin	7	6	10	8	7	8

In SCD, elevated alkaline phosphatase (ALP) is considered an indicator of bone disease or cholestasis (blockage/reduction of bile flow to the liver) (Brody et al., 1975; Kotila et al., 2005). We observed significantly higher ALP levels in HbSS mice at 4 and 5 months of age when compared to HbAA mice (Fig. 5C, *P*=0.0033 and *P*=0.0216, respectively). Additionally, we noted differences in ALP levels between sexes (*P*=0.0063). However, due to the relatively low numbers of mice examined per group, it is difficult to further interpret the trends in ALP levels over time.

Total bilirubin accounts for both direct and indirect bilirubin, i.e. both the fraction that has been processed by the liver and the fraction produced after RBC-breakdown. Total bilirubin was elevated in HbSS mice and was stable over time (Fig. 5D, *P*<0.0001 versus HbAA). Similar observations were made for direct bilirubin; however, for males, counts were somewhat higher than for females (Fig. S1F, *P*=0.0192).

Ferritin, which in SCD is typically considered an indicator of haemolysis (Fig. 5E), was significantly elevated in HbSS mice overall (*P*<0.0001), although not quite as pronounced at the 4- and 5-month timepoints.

### Kidney, heart and brain measures

**Fig. 6.**
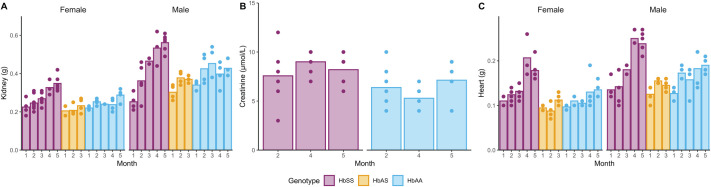
**Kidney- and heart-related measures by age and genotype.** The bars indicate mean values, while dots show the observation from each individual animal. (A) Kidney weight (g), (B) Plasma creatinine (µmol/l) and (C) heart weight (g). Comparisons between genotypes, sex, and time for each parameter were made with a heteroscedastic ANOVA with adjustments for multiple tests (Tukey method), for corresponding *P*-values, consult Table S6. Exact sample size:

Overall, when comparing between the three genotypes, kidney weights (Fig. 6A) did not reach statistical significance, but male mice had significantly heavier kidneys (*P*<0.0001, for *P*-values refer to Table S6). Furthermore, for HbSS mice, months 4 and 5 were significantly higher than month 1 (*P*=0.0024 and *P*<0.0001, respectively). The kidney-to-body weight ratio (Fig. S4A) was, however, stable over time, with significant differences observed only between HbAS and HbSS (*P*=0.0112) and between male and female mice (*P*<0.0001).

**Table:** 

Parameter	HbSS (*n*)					HbAS (*n*)			HbAA (*n*)				
Month	1	2	3	4	5	1	2	3	1	2	3	4	5
Kidney, heart	8	15	8	6	12	6	8	9	6	8	6	8	8
Creatinine	NA	7	NA	6	10	NA	NA	NA	NA	8	NA	7	8

Creatinine levels showed significant differences between HbSS and HbAA (*P*=0.0021) but appeared stable over time (Fig. 6B). The average levels for both genotypes fall within the literature reference ranges for C57Bl/6 mice (8.8±13.2 µmol/l) (Mazzaccara et al., 2008).

Heart weight (Fig. 6C) was increased in HbSS mice compared to HbAA (*P*=0.0099) and was significantly higher for months 4 and 5 compared to month 1 (*P*<0.0001, for both comparisons). Also, sex differences were observed as male mice had significantly heavier hearts compared to females (*P*<0.0001). Furthermore, the relative heart weights (Fig. S4B) were significantly increased for HbSS mice, consistent with published data (*P*<0.0001 for both HbAA and HbAS) (Menon et al., 2022) and were significantly higher in males (*P*<0.0001 versus females).

Finally, there were no overall differences in brain weight between any genotypes (Fig. S4C). The brain-to-body weight ratio decreased as the animals aged and was consistently larger in females than males (Fig. S4D), most likely driven by the animals' body growth and higher body weight in males.

### Lung weights and endothelial activation markers are only mildly affected by the sickling phenotype

**Fig. 7.**
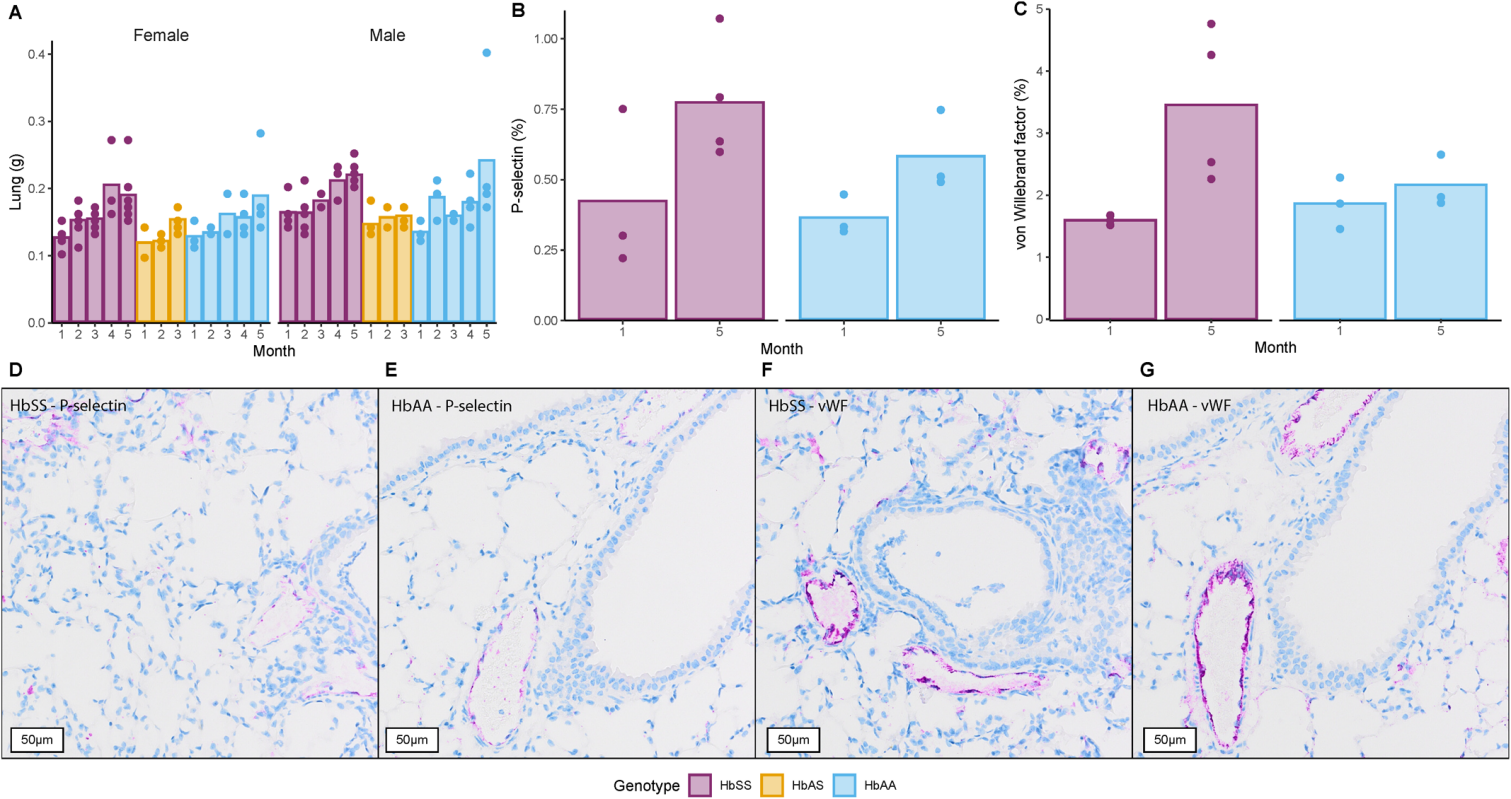
**Pulmonary parameters and immunohistochemical analysis by age and genotype.** Each bar denotes the mean value, while individual animals are shown as dots. (A) Lung weight (g). (B) Quantification of P-selectin-positive area (%) in lung tissue. (C) Quantification of vWF positive area (%) in lung tissue. (D,E) Immunohistochemical staining for P-selectin in lung sections from HbSS and HbAA genotypes, respectively (15x). (F,G) Immunohistochemical staining for vWF in lung sections from HbSS and HbAA genotypes, respectively (15x). Comparisons between genotypes, sex, and time for each parameter were made with a heteroscedastic ANOVA with adjustments for multiple tests (Tukey method) for *P*-values, refer to Table S7. Exact sample size:

Lung weights (Fig. 7A) increased as mice grew and no differences between them were observed, apart from a modest elevation in males (*P*=0.0011, for *P*-values refer to Table S7). Lung-bodyweight ratio (Fig. S4E) remained constant over time and overall, a small increase in HbSS compared to HbAS was detected (*P*=0.0183) as well as sex differences (*P*=0.0275).

**Table:** 

Parameter	HbSS (*n*)					HbAS (*n*)			HbAA (*n*)				
Month	1	2	3	4	5	1	2	3	1	2	3	4	5
Lung	8	15	8	6	12	6	8	9	6	8	6	8	8
P-selectin, vWF	3	NA	NA	NA	4	NA	NA	NA	3	NA	NA	NA	3

P-selectin in the lungs (Fig. 7B) was stained and quantified in the same way as for liver, and overall, no significant differences between genotypes were observed. Despite a limited number of observations, a slight increase in P-selectin between months 1 and 5 for the sickling genotype reached statistical significance (*P*=0.0457). P-selectin staining was primarily observed surrounding the vessels (Fig. 7D,E).

The percentage of the total lung tissue stained positive for vWF (Fig. 7C) was higher in month 5 for HbSS compared to month 1 (*P*=0.0116). Overall, there was approximately a 4-fold larger area stained for vWF compared to P-selectin, even though both are mainly expressed in endothelial cells. vWF staining can be observed mainly around the vessels in a similar pattern as P-selectin but with increased intensity and stained area (Fig. 7F,G).

## DISCUSSION

Animal models of disease have the potential to provide valuable insights into pathophysiology, and thorough characterisation of the models is likely to improve translatability of outcomes from preclinical studies into the human clinical setting. The presented dataset provides insights into the clinical presentation and welfare of the Townes mouse model of SCD, across different ages (from 4 weeks to 22 weeks) and genotypes (HbSS, HbAS and HbAA). We evaluated haematologic, biochemical and histological parameters and developed a new tool to quantify different categories of liver tissue in H&E-stained liver sections. In addition to analysing development with increasing age, we investigated potential sex differences for each parameter, since sex has been identified as a modifier that can have a differential impact on sickle cell patients in disease manifestations and laboratory measures (Masese et al., 2021).

Despite their disease, in our hands, the Townes HbSS mice gained weight at a rate similar to that of wild-type mice. As expected, spleen size in HbSS mice was significantly elevated and increased further throughout the study, whereas for the other genotypes the spleen weight did not increase. However, the spleen-to-body weight ratio for HbSS mice remained stable, showing that the spleen accounted for a constant fraction of the body weight as the mice grew. Although the enlarged spleen is likely to be a consequence of extramedullary haematopoiesis, abnormally high erythropoiesis and sequestration of sickled RBCs; spleen size in this mouse model is not considered representative of SCD patients. While splenomegaly can occur in people with SCD, it will often undergo progressive atrophy, known as autosplenectomy. Repeated episodes of infarction and necrosis can cause splenic function loss in SCD patients (El Hoss and Brousse, 2019; Brousse et al., 2014).

Both in humans and mice, the bone marrow serves as the primary site of erythropoiesis; however, extramedullary haematopoiesis also occurs in SCD. Although in healthy individuals the spleen serves as an accessory haematopoietic site for mice as well as humans, the contribution of the spleen to erythropoiesis is significantly more pronounced in mice (Schubert et al., 2008). With regard to the spleen architecture, Townes mice may be more representative of paediatric SCD, since during foetal development in humans, in addition to the bone marrow, the spleen and liver also contribute significantly to the production of RBCs (Kim, 2010). Numerous publications in SCD patients have indicated that splenomegaly occurs early in life but is subject to regional variability and is influenced by genetic and environmental factors, including prevalent infections and co-inherited conditions (Brousse et al., 2014; Tubman and Makani, 2017; Nardo-Marino et al., 2022; McCarville et al., 2011).

Furthermore, the observed difference between the enlarged spleen in the mouse model and commonly reduced spleen in humans could be due to the relative youth of the mice. We were not able to investigate whether splenic sequestration occurred in older mice, nor find any publications reporting on longitudinal spleen weight changes or documenting spleen weight in older Townes mice.

Anaemia and haemolysis are characteristic features of SCD, and as expected, lower haemoglobin, RBC and MCHC were observed in HbSS compared to the other two genotypes. These results are consistent with literature showing that HbSS mice (and SCD patients) have lower levels (Wu et al., 2006; Charrin et al., 2016; Alvarez-Argote et al., 2023; Macharia et al., 2018) whereas HbAA and HbAS are on par with normal mouse values (Stahl et al., 2018; Mazzaccara et al., 2008; Santos et al., 2016). As a novel insight, we show that these parameters did not change significantly during the 5-month observation period in this study, implying that in the Townes mice, the anaemia-related aspect of the disease does not progress significantly during the first 5 months of life. This indicates that within this age interval, studies can be conducted without major impact from changes in the disease stage.

We observed lower platelet counts in HbSS mice, which is consistent with data published by Charrin and colleagues (2016), but differs from Alvarez-Argote and colleagues' paper (2023), where they observed and increase in platelet counts for HbSS mice. We also discovered differences between sex, which has not been previously reported in SCD mice, although such variations have been documented in wild-type mice (Mazzaccara et al., 2008; Stahl et al., 2018).

In our study, we found that HbSS mice have a higher MCV compared to the other genotypes, suggesting an elevated proportion of immature RBCs in circulation (as immature RBCs are larger than mature RBCs) (Aslinia et al., 2006). The scarcity of irreversibly sickled cells on blood smears (Fig. S5, representative images) implies that those cells only have a minor impact on the MCV. The observed increase in MCV in the murine model, which contrasts with the typical decrease seen in humans with SCD, may be caused by species-specific differences in the compensatory responses to anaemia (Glader et al., 1979; Kamimura et al., 2024). Whereas human RBCs tend to become denser and more dehydrated in SCD, resulting in a reduced MCV, the response in mice may be dominated by a rise in reticulocyte counts (Almeida et al., 2024). Interestingly, even for HbSS mice, the observed MCV was within the published range for normal C57Bl/6 mice (Santos et al., 2016; Stahl et al., 2018).

Reticulocyte counts accounted for 50% of the total RBCs in HbSS mice, supporting the observation of increased MCV compared to the other genotypes. Even though the decreasing ­trend in total reticulocyte numbers for HbSS mice was statistically significant, this was not the case when reticulocytes were expressed as a percentage of total RBCs, indicating that it may not have biological significance. We also analysed the different reticulocyte fractions, and as expected, HFR, the most immature fraction, was elevated in HbSS. This finding is consistent with the continuous haemolysis and compensating erythropoiesis characteristic of SCD pathophysiology.

The final haematology parameter, haematocrit (HCT), was comparable between the genotypes in our study, with HbSS at 34.3±5.3, HbAS at 37.6±4.5 and HbAA at 36±6.1. These levels align with data from The Jackson Laboratory for 8-week-old Townes mice for HbSS but are lower for the other two genotypes (The Jackson Laboratory, 2023). In contrast, our results are higher than Charrin and colleagues' data from mice aged 12 to 18 weeks (HbSS 26.8±5.1, HbAS 34.3±3.4 and HbAA 31.8±7.3) (Charrin et al., 2016). It is important to note that the differences in these numbers may also be due to different instruments being used. The HCT in our sickling mice, despite lower RBC counts and haemoglobin levels, may be due to the higher MCV. The increased volume of cells, which may be attributed to a greater presence of immature RBCs (approximately 50% of total RBCs are reticulocytes in HbSS), is likely to be counteracting the overall lower RBC counts, resulting in a ‘normal’ HCT. However, the observed HCT for Townes mice is uniformly lower compared to reference values for C57Bl/6 mice. This may be explained by the mixed C57Bl6/129 genetic background of Townes mice, as background strain is known to influence HCT (Mazzaccara et al., 2008). Additionally, age-related variation could account for discrepancies in HCT levels reported in the literature, with older mice tending to have lower HCT (Flurkey et al., 2007). Consistent with this, we also observed a minor decrease in HCT as age increased in both HbSS and HbAA genotypes.

We observed significantly elevated peak thrombin levels and endogenous thrombin potential in HbSS mice compared to wild-type Townes mice, aligning with previous studies and consistent with the hypercoagulable state reported in SCD (Sparkenbaugh et al., 2023). This may be due to increased factor VIII and other haemostatic alterations, such as elevated tissue factor and activated platelets, which are known to contribute to the prothrombotic state in human SCD (Conran and De Paula, 2020).

We report that WBC counts (both in total as well as lymphocytes, neutrophils, and monocytes considered separately) were significantly increased in HbSS mice compared to the non-sickling genotypes. This finding aligns with the association of SCD with an ongoing state of inflammation and endothelial activation, described as sterile inflammation (Conran and Belcher, 2018). Male mice had higher overall WBC counts as well as higher neutrophil and monocyte counts, but no sex differences were observed in lymphocyte counts although lymphocytes are the most abundant cell type in the WBC population (75-90%) (Mestas and Hughes, 2004). Within the same genotype, some leukocyte populations appeared to change during the course of the study, in some cases reaching statistical significance; however, variability was high, and no clear directionality was deduced.

Patients with SCD have been reported to exhibit elevated levels of cytokines such as TNF-α, IL-1β, IL-6 and IL-8. Specifically, TNF-α and IL-6 levels have been observed to increase during VOCs (Taylor et al., 1995; Sarray et al., 2015; Lanaro et al., 2009). In agreement with patient data, we found that IL-6, IL-2 and TNF-α were significantly increased in HbSS mice compared to HbAS and IL-10 was elevated in HbSS mice compared to HbAA. But overall, except for IL-5, which was elevated in all genotypes, the measured levels were still within the normal range of control mice [(MSD) 2013].

Examining additional organs for damage, we found that HbSS mice had enlarged livers compared to other genotypes. Additionally, there was a significant increase in liver weights and liver-to-body weight ratios beginning at month 3, after the main growth phase of the liver, accompanied by the characteristic ‘nutmeg’ appearance and histopathological evidence, which is strongly indicative of liver damage (Moreno-Carranza et al., 2018). These findings are consistent with the known pathophysiology of SCD, where sickling of RBCs can obstruct blood vessels in the liver, leading to liver damage and enlargement.

In pursuit of a quantitative and unbiased assessment of liver damage, we trained an AI-based algorithm to analyse H&E-stained liver images, speculating that this new method could enhance the traditional scoring methods. Our results showed that HbSS mice consistently had elevated levels of RBC clots, necrotic tissue, and diseased liver tissue compared to the other genotypes. Consequently, HbSS mice had a significantly lower percentage of normal liver tissue compared to HbAS and HbAA mice. Increased levels of diseased liver tissue, which are likely to indicate acute lesions, were observed from month 2 and onwards in HbSS mice. Necrotic tissue, which could be indicative of chronic damage, was significantly elevated in month 2 compared to the first month, but not in later months. Since liver growth was still ongoing throughout the study (as illustrated by the increasing liver weight), tissue damage may to some extent be masked by the growth (Moreno-Carranza et al., 2018).

These histological findings from the AI-based tissue classifier align with conventional histopathological observations, which detected focal necrosis, surrounded by inflammatory cells (probably caused by reduced blood flow and subsequent ischaemic necrosis), RBC accumulation in sinusoids and larger vessels, and frequent haemosiderin deposits in Kupffer cells of HbSS mice. These pathologies, observed only in sickling mice, are consistent with literature both in Townes mice and SCD patients (Manci et al., 2014; Trucas et al., 2023; Saeed et al., 2022; Nguyen et al., 2014).

Endeavouring to further elucidate the pathophysiology of SCD in this model, we investigated vWF and P-selectin distribution in the liver. vWF is involved in blood clotting, and patients with SCD have higher vWF plasma levels, suggesting that vWF may be involved with the vascular complications associated with SCD (Nwagha et al., 2022). P-selectin is involved in the interaction between white blood cells, platelets and endothelial cells during the inflammatory response. In SCD, P-selectin is chronically overexpressed and has been linked to VOCs (Solovey et al., 1997; Karki and Kutlar, 2021). In HbSS mice, both P-selectin and vWF in liver were found elevated at month 5 compared to HbSS mice at 1 month, as well as HbAA mice. Although our sample size is small, this finding aligns with the histopathological findings of less necrotic and diseased liver tissue at 1 month. We hypothesise that at 1 month, sufficient damage to manifest in chronic alterations may not yet have occurred, and/or that liver growth may be counteracting or obscuring tissue damage in these maturing animals. In future studies it could be considered to assess additional endothelial activation markers such as VCAM-1 and E-selectin to provide a more comprehensive understanding of endothelial activation in these mice.

Even though the levels of AST, ALT, ALP and ferritin are consistently elevated in HbSS mice compared to HbAA mice, the highest levels were observed in younger mice. Although we have no way to verify experimentally, we speculate that this may be due to the lengthy overseas transportation and subsequent acclimatisation of the mice to the new animal facility, which could be a stressor strong enough to trigger such metabolic changes in HbSS animals. During the following months of tranquillity (after acclimatisation), the disease appears to stabilise leading to a gradual decline of some of those parameters. Direct and total bilirubin, indirect markers of haemolysis, were elevated in HbSS mice and no differences over time were observed, supporting that the phenotype of haemolysis and regenerative anaemia was unchanged throughout the study. Overall, these observations are consistent with the known pathophysiology of SCD in the liver and support the potential of AI-based image analysis techniques in studying liver pathology.

We also studied the kidney, heart and brain; overall, there were no significant differences in kidney and brain weights between genotypes. Creatinine measures showed significant differences between sickling and HbAA mice, which could indicate an effect of the disease on the kidney; however, since both groups were still within normal reference ranges for healthy mice, it remains unclear whether those differences are biologically relevant.

Heart weight and heart-to-body weight ratio were elevated overall in HbSS mice compared to HbAA and increased during the study in both male and female at 4 and 5 months of age, indicating that the lower haemoglobin content (and consequently oxygen carrying capacity) of the blood may increase the workload for the heart. Morphologically, no substantial differences were observed besides sickling cells in the cardiac vessels of HbSS mice. However, in patients it has been observed that there is an increase in left ventricular mass to compensate for the ongoing chronic anaemia (Gladwin and Sachdev, 2012; Lester et al., 1990), and our data support that a similar adaptive response occurs in HbSS mice. It is also noteworthy that both kidney and heart weights were increased in males, which is likely a reflection of their overall body weight increase, since no overall differences in their organ ratios were observed.

Finally, histological examination of Townes sickling homozygotes' lungs revealed sickle cells accumulating in the capillaries, potentially causing obstruction. However, there was no evidence of deterioration during the study. Both P-selectin and vWF were found to be moderately increased in HbSS mice at month 5 compared to month 1, but interpretation is challenged by the limited sample size.

In conclusion, the presented dataset provides valuable insights into the pathophysiology of SCD in this model, and its relevance and value for investigation of different SCD features. HbSS Townes mice presented SCD characteristics including anaemia, haemolysis and organ damage (as observed by histology and metabolic markers). Additionally, we introduced a new tool to automate and quantify liver H&E staining, offering quantitative and unbiased assessment of liver damage. Interestingly, the study also showed differences between sexes for some leukocyte populations, ALP and platelet counts, underlining the need for appropriate controls in preclinical studies.

The results indicate that the anaemia-related aspect of the disease does not progress significantly during the first 5 months of life, providing a time span within which studies can be conducted without necessarily being impacted by changes in disease stage, and without compromising animal welfare.

Our comprehensive study of the Townes mouse model sheds new light on the utility of this model in advancing our understanding of SCD pathophysiology. Acknowledging the inherent limitations of any model to fully reproduce the human condition, we here show that Townes mice recapitulate haematological, biochemical, and histopathological aspects of the disease, implying their utility for study thereof. The novel AI-based analysis technique adds to traditional methods a more quantitative aspect, aiding evaluation of liver damage. Overall, our findings provide a detailed description of the clinical presentation of Townes mice from youth to maturity, providing a valuable reference framework for future research.

## MATERIALS AND METHODS

### Mice

Townes mice were acquired from Jackson Laboratories, Bar Harbour, USA (JAX stock #013071) (Wu et al., 2006). Male and female Townes mice were group-housed under standardized conditions (21°C, 60% relative humidity, a 12-h/12-h light/dark cycle, and *ad libitum* access to food and water) in environmentally enriched cages. Randomisation methods were employed to allocate animals to experimental groups, ensuring unbiased distribution. Data from two studies are presented in this manuscript. The first study followed Townes mice from 4 weeks of age until 12-13 weeks of age and the second study followed Townes mice from 8 weeks of age until 22 weeks of age to assess disease changes into maturity. The results are presented as a meta-analysis of both studies. Mice were weighed twice weekly and observed daily. Experiments were approved and performed according to guidelines by the Novo Nordisk Ethical Review Council and the Danish Animal Experiments Inspectorate, The Danish Ministry of Food, Agriculture, and Fisheries.

The minimum sample size of eight was determined based on the mean and standard deviation of RBC counts from published data by Jackson Laboratory (The Jacskon Laboratory, 2023). Assuming a significance level of 5%, this sample size provides a 95% power to detect a biologically relevant 20% change [InVivoStat v4.9, (Clark et al., 2012)].

### Peripheral blood samples and blood smears

Mice were anaesthetised with isoflurane [induction at 5% isoflurane and maintenance anaesthesia at 2% (70% O_2_ and 30%N_2_O)]. Terminal blood samples were collected from the retrobulbar venous plexus using a capillary tube (25 µl EDTA #78213, Vitrex) into an EDTA-coated 1.5 ml Eppendorf tube (2MG/ML EDTA-K2 #041-TOM-14C, Milian). Briefly, 1.5 µl of whole blood were used for blood smears, and stained with Hemacolor^®^ staining set (#111661, Merck) according to the manufacturer’s instructions.

### Complete blood counts

25 µl of EDTA-blood were diluted into 100 µl of CELLPACK buffer (#834-0011-10, Sysmex) and run within the following 4 h on a Sysmex XT-2000i Hematology Analyzer (Kobe, Japan), using the function ‘Capillary mode’.

The following parameters were obtained from each sample: RBC counts, Hb, HCT, MCV, MCH, MCHC, platelets, reticulocytes, WBC count and WBC differential percentages and absolute counts. Since Townes mice have a mixed genetic background of C57Bl/6 and 129 mice, and genetic ratios in mixed-background strains are often unpredictably variable, we selected C57Bl/6 literature values as normal reference controls due to their consistent documentation and availability for various ages, providing a stable baseline for comparison.

### Metabolic analysis

EDTA-blood was centrifuged at 4000 ***g*** for 5 min. The top plasma layer (100 µl) was transferred to COBAS tubes and frozen at −80°C until analysis. Samples were run on a COBAS 6000 c501 (Roche Diagnostics A/S). The following metabolic parameters were measured: ALT, AST, ALP, direct and total bilirubin, creatinine and ferritin.

### Tissue sampling and processing

After terminal blood sampling, mice (still under anaesthesia) were euthanized by exsanguination through an incision of the abdominal aorta. The following organs were removed and weighed: spleen, kidneys, liver, heart, brain and lungs. All organs were fixed in 10% neutral buffered formalin (VWR International AB, Sweden) for 24 to 48 h. Specifically, the lungs were fixed with 10% neutral buffered formalin by inserting a blunt needle into the trachea and filling the lungs with approximately 0.5 ml neutral buffered formalin. All organs were dehydrated via graded concentrations of ethanol (Kemetyl A/S, Køge, Denmark), transferred to xylene (Applichem, GmbH, Darmstadt, Germany) and embedded in paraffin.

### Histochemistry and immunohistochemistry

Sections of the liver and spleen of 3 µm thickness were cut and stained with H&E (MSH80-2,5L and HT110208-2,5L; both from Sigma-Aldrich, USA).

Immunohistochemical staining for P-selectin in liver and lung sections was performed on a Ventana Discovery Ultra platform (Roche). The sections were heated at 72°C for 12 min, and then the heat-induced epitope retrieval was performed in Cell Conditioning Solution 1 (#950-224, Roche) at 95°C for 24 min. Endogenous peroxidase was blocked by applying Discovery inhibitor (#760-4840, Roche) for 12 min. Afterwards, the unspecific binding of the primary antibody was blocked in TNB buffer (Roche) for 20 min. A goat anti-P-selectin antibody (#AF737, R&D Systems) was used as the primary antibody at a concentration of 0.2 µg/ml diluted in TNB buffer (Roche) and applied to the slides at 37°C for 60 min. For the detection, UltraMap horse radish peroxidase anti-goat (#6607241001, Roche) was used and applied to the sections for 20 min at 35°C. After, a purple chromogen (Discovery Purple Kit, Roche) was applied for 32 min. Finally, the slides were counterstained with Haematoxylin (#790-2208, Roche) for 4 min and bluing reagent (Roche) for 4 min. Cover glasses were mounted with Pertex (Sigma-Aldrich). Negative controls did not include the primary antibody, which was replaced by a goat isotype control (#005-000-003, Jackson ImmunoResearch) at the same concentration as the primary antibody.

Immunohistochemical staining for vWF was performed as well in a Ventana Discovery Ultra Platform (Roche) following the same steps as described above. The only differences were the primary antibody step, which was a rabbit anti-vWF (#A0082, Dako, Santa Clara, USA) for 60 min at 37°C at a dilution of 0.5 µg/ml in TNB buffer, and the detection step, in which first anti-rabbit-HQ (Roche) was used at 37°C for 24 min followed by 16 min of anti-HQ horse radish peroxidase (Roche). For negative controls, a rabbit IgG isotype control was used (#011-000-003, Jackson ImmunoResearch) at the same concentration as the primary antibody, 0.5 µg/ml.

A digital whole-slide image of each liver and lung section was generated by scanning the sections on a NanoZoomer 2.0HT digital slide scanner (Hamamatsu) according to the manufacturer’s instructions.

### Quantification of liver tissue types with HALO AI image analysis software

Whole-slide images of liver tissue H&E sections were analysed with HALO AI 3.5 (Indica Labs). Two different AI algorithms were trained and used for this purpose.

First, an AI-based algorithm (Random Forest) for identification of tissue was trained. Manually annotated areas of tissue and background were drawn in three different sections, representing Townes mice both with HbAA and HbSS genotype. Minimum object size was adjusted to 1,000,000 µm to select the whole liver sections and only six annotations were needed for the first classifier to work.

Secondly, a DenseNet V2 classifying algorithm was trained to classify the tissue in five different categories: background, normal healthy liver tissue, RBC clot, infarcted area (necrotic tissue) and diseased liver tissue (which included areas with inflammation, extramedullary proliferation and bile duct proliferation). This was done by manually drawing annotations of each category in 15 different tissue sections from 15 different randomly selected Townes mice (both HbSS and HbAA mice genotypes). The performance of the algorithm was assessed by several visual inspections during the training process. Erroneous classification of tissue was corrected by manually adding accurate annotations to the training set and followed by retraining the algorithm. In total, 674 annotations were made: 254 annotations of background, 39 annotations of normal liver, 181 RBC clots, 32 infarcted and 168 diseased liver tissue. The classification by the final algorithm was checked by an experienced pathologist and found to be valid.

The trained AI-based algorithms were then used for analysis of all liver sections. First the Random Forest classifier was used to identify areas with liver tissue, and then the DenseNet V2 was run on the tissue layer. The different outputs were plotted as percentage of tissue derived from the Random Forest classifier.

### Statistical analysis

Data presented in the text are expressed as mean±standard deviation (s.d.). In figures, data are shown as individual values with the mean indicated. Statistical analysis was conducted using the statistical software R (R Core Team, 2022). Comparison between genotypes, sex, and time for each parameter were made with a heteroscedastic ANOVA model. If there were differences between sex, data are plotted separately for males and females. *P*-values and 95% confidence intervals were adjusted for multiple testing using the max-*t*-test method, also known as the Tukey method.

## Supplementary Material

10.1242/biolopen.061828_sup1 Supplementary information
