# Supplementary material for: From early development to maturity: a phenotypic analysis of the Townes sickle cell disease mice
Source: Biol Open. 2025 Feb 6;14(2):bio061828. doi: 10.1242/bio.061828 (PMC11832121; doi:10.1242/bio.061828)
Supplement: Supplementary information [file biolopen-14-061828-s1.pdf]

Biology Open (2025): doi:10.1242/bio.061828: Supplementary information

Table S1. P-values obtained from ANOVA comparisons between genotypes, sex and time, with adjustment for multiple tests using the Tukey method for parameters displayed in Fig. 1.

**FIGURE 1**		**Genotypes**			**Sex**
Parameter		**HbSS vs HbAA**	**HbSS vs HbAS**	**HbAA vs HbAS**	**Male vs Female**
Last body weight (g)		0.9170	0.7150	0.9060	<0.0001
Spleen (g)		<0.0001	<0.0001	0.1800	0.0558
Haemoglobin (mmol/L)		<0.0001	<0.0001	0.1020	0.9990
Platelets (10^9/L)		<0.0001	<0.0001	<0.0001	<0.0001
		**Month 1 vs**			
		**Month2**	**Month3**	**Month4**	**Month5**
Last body weight (g)	HbSS	0.5670	0.0193	<0.0001	<0.0001
	HbAS	0.2367	0.0381		
	HbAA	0.8575	0.0126	0.0340	0.0121
Spleen (g)	HbSS	0.0329	<0.0001	<0.0001	<0.0001
	HbAS	0.9970	0.9858		
	HbAA	0.9837	1.0000	1.0000	0.9088
Haemoglobin (mmol/L)	HbSS	0.6664	0.7813	0.2493	0.6321
	HbAS	0.9913	0.2926		
	HbAA	0.1228	0.9976	0.0463	0.0323
Platelets (10^9/L)	HbSS	0.0726	0.9592	0.9872	0.9999
	HbAS	0.9285	0.7740		
	HbAA	0.1103	0.8563	0.2091	0.4511

Biology Open • Supplementary information

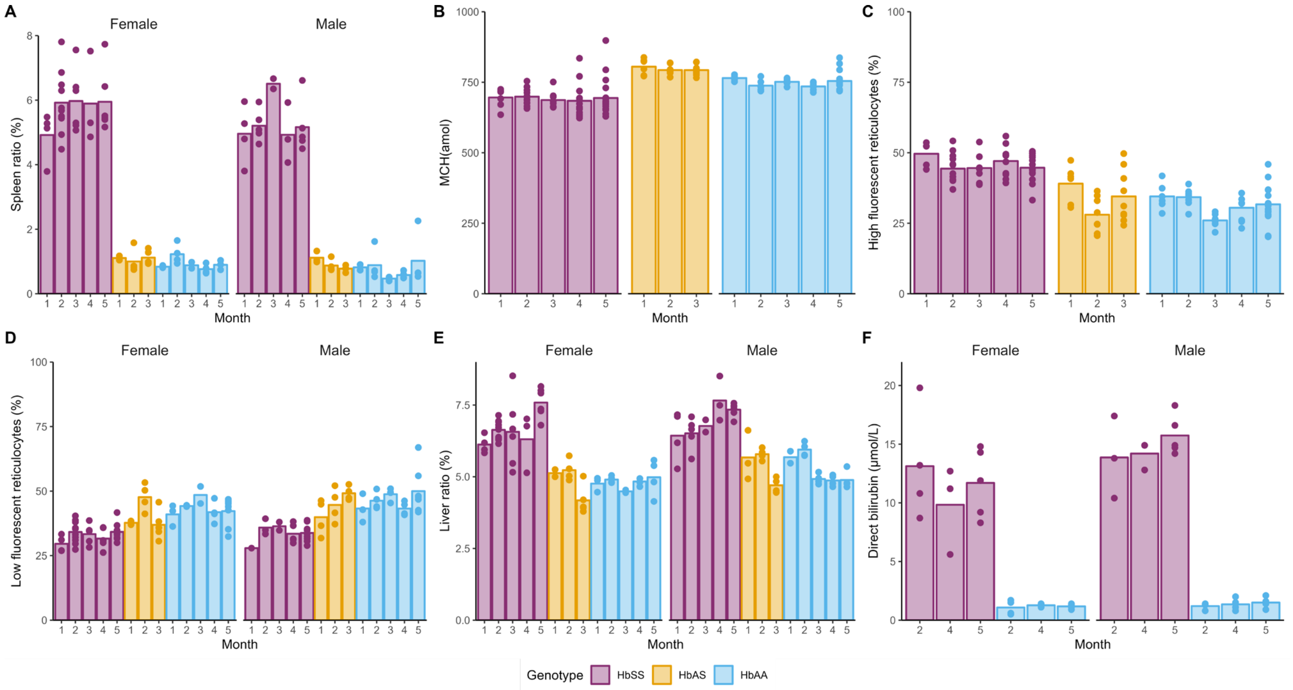

Fig. S1. Organ ratios, haematological and biochemical parameters separated by age and genotype. Data are presented as mean values (bars) and individual mice represented by dots. (**A**) Ratio of spleen weight to body weight (%). (**B**) Mean corpuscular haemoglobin (MHC, amol). (**C**) Immature reticulocyte fraction displayed as high fluorescent reticulocytes (%). (**D**) Low fluorescence reticulocytes (%), representing the less immature fraction of reticulocytes. (**E**) Ratio of liver weight to body weight (%). (**F**) Direct bilirubin measurements (µmol/L). Genotype designations are indicated by colour: HbSS (purple), HbAS (yellow), HbAA (blue).

Parameter	HbSS (n)					HbAS (n)			HbAA (n)				
Month	1	2	3	4	5	1	2	3	1	2	3	4	5
Spleen ratio (%), liver ratio (%)	8	15	8	6	12	5	8	9	5	8	6	8	8
MCH (amol)	8	15	8	14	20	6	8	9	6	8	6	8	16
HFR (%), LFR (%)	5	13	7	11	17	6	8	9	6	8	6	8	15
Direct bilirubin (µmol/L)	NA	7	NA	6	10	NA	NA	NA	NA	8	NA	7	8

Table S2. *P*-values obtained from ANOVA comparisons between genotypes, sex and time, with adjusting for multiple tests using the Tukey method for parameters displayed in Fig. 2.

FIGURE 2		Genotypes			Sex
Parameter		HbSS vs HbAA	HbSS vs HbAS	HbAA vs HbAS	Male vs Female
Red blood cells (10^{12}/L)		<0.0001	<0.0001	0.9630	0.7570
Haematocrit (%)		0.2220	0.5130	0.9920	0.8480
MCV (fL)		<0.0001	<0.0001	0.6670	0.1380
MCHC (mmol/L)		<0.0001	<0.0001	<0.0001	0.5560
Reticulocytes (10^{12}/L)		<0.0001	<0.0001	0.9760	0.6770
Reticulocytes (%)		<0.0001	<0.0001	0.9606	0.7092
		Month 1 vs			
		Month2	Month3	Month4	Month5
Red blood cells (10^{12}/L)	HbSS	0.6253	0.8327	0.3606	0.6608
	HbAS	0.9907	0.5252		
	HbAA	0.3972	1.0000	0.2137	0.1201
Haematocrit (%)	HbSS	0.1362	0.4931	0.0215	0.0220
	HbAS	0.9863	0.2235		
	HbAA	0.2632	0.8152	0.0512	0.0294
MCV (fL)	HbSS	0.7900	0.9726	0.4308	0.0300
	HbAS	0.9013	0.5513		
	HbAA	0.9925	0.5094	0.7557	0.9282
MCHC (mmol/L)	HbSS	0.8831	0.9999	0.9049	0.1588
	HbAS	0.9869	0.5525		
	HbAA	0.9845	0.4285	0.9953	0.9571
Reticulocytes (10^{12}/L)	HbSS	0.3318	0.8647	0.0031	0.0038
	HbAS	0.9611	0.9895		
	HbAA	0.9650	0.9615	0.7095	0.6719
Reticulocytes (%)	HbSS	1.0000	0.9997	0.5356	0.2303
	HbAS	0.9786	0.9539		
	HbAA	0.9998	0.9915	0.9610	0.9629

Biology Open • Supplementary information

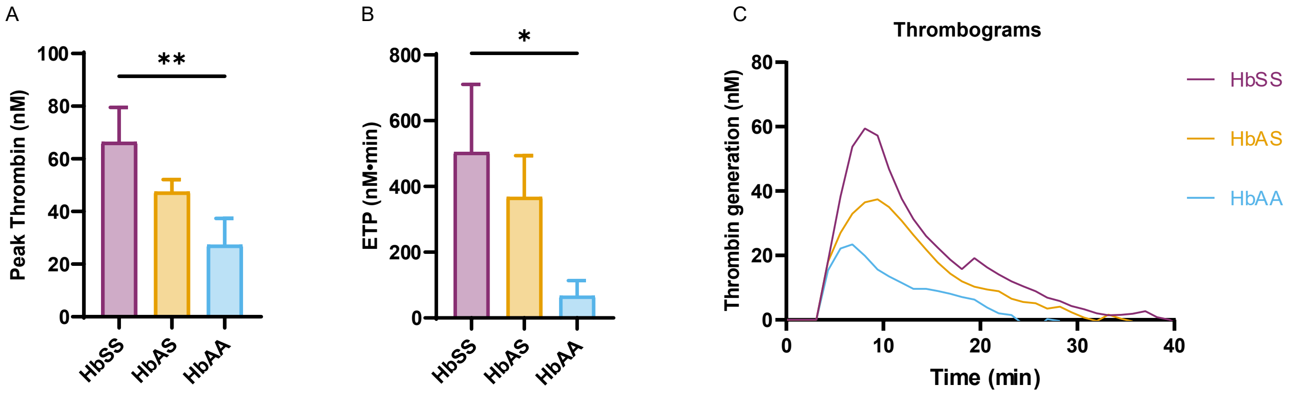

Fig. S2. Thrombin generation in 3-month-old mice by genotype. Thrombin generation was assessed in citrated whole blood triggered with $CaCl_2$. Measurements were conducted on a Flouroskan Ascent (Thermo Scientific) with Ascent Software. Bars represent mean values with error lines indicating standard deviation (s.d.). Whole blood samples were obtained from HbSS (n=8), HbAS (n=2) and HbAA (n=2) mice for the assay. (**A**) Peak thrombin levels (nM). (**B**) Endogenous thrombin potential (ETP, nM·min). (**C**) Thrombogram depicting mean thrombin generation by time and genotype.

Biology Open • Supplementary information

Table S3. *P*-values obtained from ANOVA comparisons between genotypes, sex and time, with adjusting for multiple tests using the Tukey method for parameters displayed in Fig. 3.

FIGURE 3		Genotypes			Sex
Parameter		HbSS vs HbAA	HbSS vs HbAS	HbAA vs HbAS	Male vs Female
White blood cells (10^9/L)		<0.0001	<0.0001	0.6460	0.0040
Lymphocytes (10^9/L)		<0.0001	<0.0001	0.4840	0.4010
Neutrophils (10^9/L)		<0.0001	<0.0001	0.2130	0.0057
Monocytes (10^9/L)		<0.0001	<0.0001	0.0639	0.0007
		Month 1 vs			
		Month2	Month3	Month4	Month5
White blood cells (10^9/L)	HbSS	0.0036	1.0000	0.0583	0.3455
	HbAS	0.9771	0.7941		
	HbAA	0.9964	1.0000	0.9986	1.0000
Lymphocytes (10^9/L)	HbSS	0.9887	0.0269	0.5250	0.7765
	HbAS	0.9879	0.8107		
	HbAA	0.9262	1.0000	0.9840	1.0000
Neutrophils (10^9/L)	HbSS	0.9999	0.8613	0.6435	0.4996
	HbAS	0.8332	0.9488		
	HbAA	0.9933	0.9973	0.9841	0.9588
Monocytes (10^9/L)	HbSS	0.5084	<0.0001	0.0043	0.0549
	HbAS	0.9769	0.9978		
	HbAA	0.9867	1.0000	0.9874	0.9998

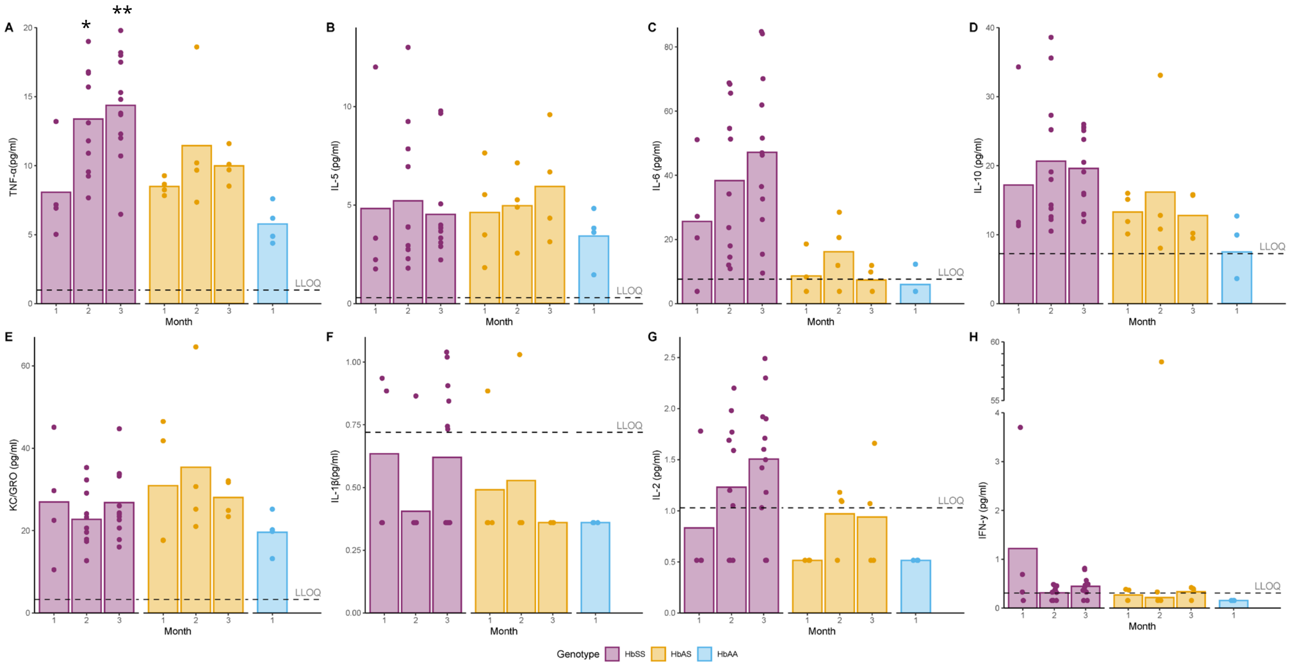

Fig. S3. Cytokine levels by age and genotype. Cytokine levels (pg/ml) in mouse plasma were analysed using the V-PLEX Plus Proinflammatory Panel 1 Mouse Kit (#K15048G, Mesoscale Discovery) according to the manufacturer's protocol. Each bar represents the mean value, with individual data points displayed as dots. (**A**) Tumour necrosis factor alpha (TNF-α). (**B**) Interleukin 5 (IL-5). (**C**) Interleukin 6 (IL-6). (**D**) Interleukin 10 (IL-10). (**E**) Keratinocyte chemoattractant/growth-regulated oncogene (KC/GRO). (**F**) Interleukin 1 beta (IL-1β). (**G**) Interleukin 2 (IL-2) and (**H**) interferon gamma (INF-γ). The dashed line indicates the lower limit of quantification (LLOQ). Values below the LLOQ are plotted as LLOQ/2. *P*-values compared to month 1, with * indicating *P*<0.05 and ** *P*<0.01. Sample size for all parameters:

	HbSS (n)			HbAS (n)			HbAA (n)
**Month**	**1**	**2**	**3**	**1**	**2**	**3**	**1**
All parameters	4	11	8	4	4	4	4

Biology Open • Supplementary information

Table S4. *P*-values obtained from ANOVA comparisons between genotypes, sex and time, with adjusting for multiple tests using the Tukey method for parameters displayed in Fig. 4.

FIGURE 4	Genotypes			Sex
Parameter	HbSS vs HbAA	HbSS vs HbAS	HbAA vs HbAS	Male vs Female
Liver (g)	<0.0001	0.0073	0.3897	<0.0001
Clot of RBC (%)	<0.0001	<0.0001	0.6030	0.8297
Necrotic tissue (%)	<0.0001	<0.0001	0.6060	0.4163
Diseased liver tissue (%)	<0.0001	<0.0001	0.4740	0.8367
Liver P-selectin (%)	0.0010	0.1510	0.6190	
Liver von Willebrand factor (%)	0.0010	0.0045	0.6190	

		Month 1 vs			
		Month2	Month3	Month4	Month5
Liver (g)	HbSS	0.4611	0.0318	<0.0001	<0.0001
	HbAS	0.6364	0.9959		
	HbAA	0.8893	0.5135	0.6142	0.4070
Clot of RBC (%)	HbSS	0.7899	0.6025	0.0125	0.9946
	HbAS		0.1488		
	HbAA	0.4268	0.9999	0.9131	0.5269
Necrotic tissue (%)	HbSS	<0.0001	0.9974	0.8535	0.9985
	HbAS		0.9078		
	HbAA	1.0000	1.0000	1.0000	1.0000
Diseased liver tissue (%)	HbSS	<0.0001	0.0015	0.0035	<0.0001
	HbAS		0.5755		
	HbAA	0.9970	0.9718	0.9928	0.9997
Liver P-selectin (%)	HbSS				<0.0001
	HbAA				0.6025
Liver von Willebrand factor (%)	HbSS				<0.0001
	HbAA				0.3558

Table S5. *P*-values obtained from ANOVA comparisons between genotypes, sex and time, with adjusting for multiple tests using the Tukey method for parameters displayed in Fig. 5.

**FIGURE 5**	**Genotypes**	**Sex**	**HbSS Month 2 vs**		**HbAA Month 1 vs**		**HbSS vs HbAA**		
Parameter	**HbSS vs HbAA**	**M vs F**	Month 4	Month 5	Month 4	Month 5	Month 2	Month 4	Month 5
Alanine transaminase (U/L)	<0.0001	0.8790	<0.0001	<0.0001	0.9997	0.9997	<0.0001	0.1105	0.0392
Aspartate aminotransferase (U/L)	0.0002	0.7600	<0.0001	<0.0001	0.9998	0.9892	<0.0001	0.3444	0.1913
Alkaline phosphatase (U/L)	0.0010	0.0063	0.3324	0.8076	0.5337	0.6509	0.4648	0.0033	0.0216
Total bilirubin (μmol/L)	<0.0001	0.0875	0.4901	0.9956	0.9995	0.9955	<0.0001	<0.0001	<0.0001
Ferritin (μg/L)	<0.0001	0.5340	<0.0001	<0.0001	0.9794	0.9351	<0.0001	0.0863	0.0076

Biology Open • Supplementary information

Table S6. *P*-values obtained from ANOVA comparisons between genotypes, sex and time, with adjusting for multiple tests using the Tukey method for parameters displayed in Fig. 6.

FIGURE 6		Genotypes			Sex
Parameter		HbSS vs HbAA	HbSS vs HbAS	HbAA vs HbAS	Male vs Female
Kidney (g)		0.8850	0.6590	0.8940	<0.0001
Creatinine (µmol/L)		0.0021			0.1198
Heart (g)		0.0099	0.0773	0.9631	<0.0001
		Month 1 vs			
		Month2	Month3	Month4	Month5
Kidney (g)	HbSS	0.7939	0.4470	0.0024	<0.0001
	HbAS	0.8962	0.8836		
	HbAA	0.7712	0.3320	0.9456	0.5414
Creatinine (µmol/L)	HbSS*			0.3634	0.7747
	HbAA*			0.5033	0.7032
Heart (g)	HbSS	0.9840	0.6824	<0.0001	<0.0001
	HbAS	0.9282	0.7671		
	HbAA	0.4418	0.5530	0.0873	0.0353

** Month 2 comparison instead of Month 1*

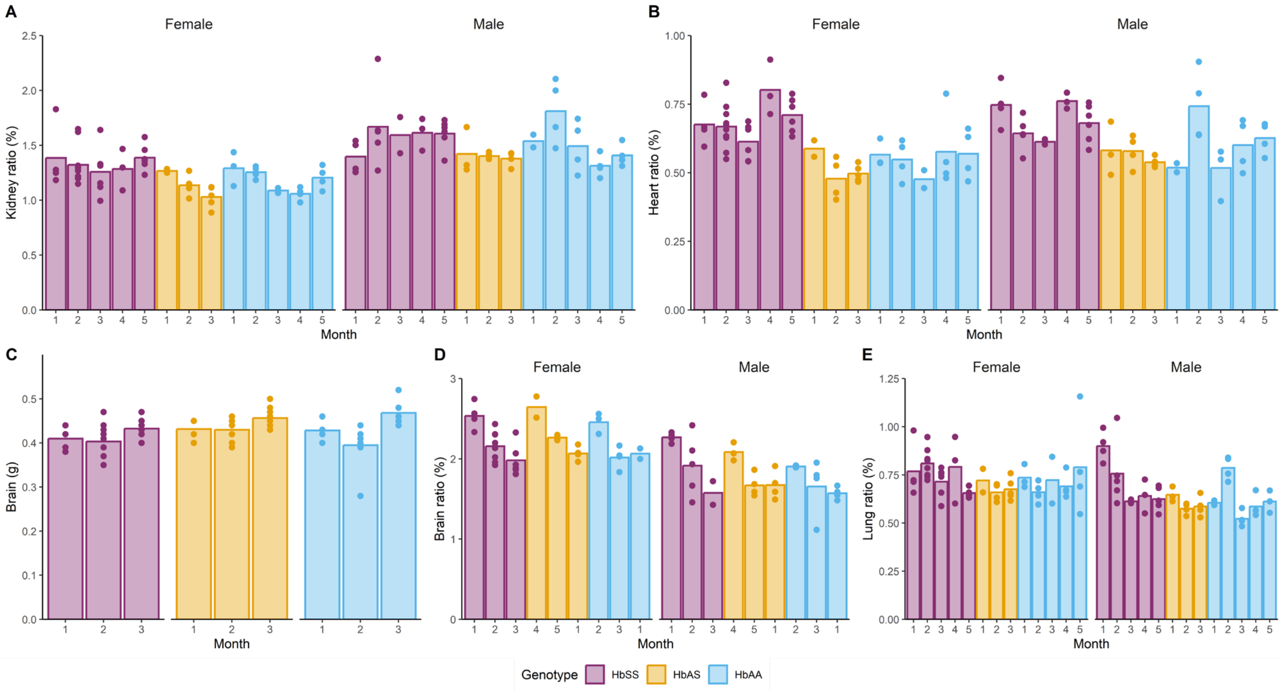

Fig. S4. Organ weights and ratios by age and genotype. Bars indicate mean values, with individual animals represented as dots. (**A**) Kidney-to-body weight ratio (%). (**B**) Heart-to-body weight ratio (%). (**C**) Brain weight (g). (**D**) Brain-to-body weight ratio (%). (**E**) Lung-to-body weight ratio (%).

Parameter	HbSS (n)					HbAS (n)			HbAA (n)				
**Month**	**1**	**2**	**3**	**4**	**5**	**1**	**2**	**3**	**1**	**2**	**3**	**4**	**5**
Kidney ratio (%), heart ratio (%), lung ratio (%)	8	15	8	6	12	5	8	9	5	8	6	8	8
Brain (g)	8	15	8	NA	NA	6	8	9	6	8	6	NA	NA
Brain ratio (%)	8	15	8	NA	NA	5	8	9	5	8	6	NA	NA

Biology Open • Supplementary information

Table S7. *P*-values obtained from ANOVA comparisons between genotypes, sex and time, with adjusting for multiple tests using the Tukey method for parameters displayed in Fig. 7.

FIGURE 7		Genotypes			Sex
Parameter		HbSS vs HbAA	HbSS vs HbAS	HbAA vs HbAS	Male vs Female
Lungs (g)		0.7080	0.4230	0.8400	0.0011
Lungs P-selectin (%)		0.2529			
Lungs von Willebrand factor (%)		0.2671			
		Month 1 vs			
		Month2	Month3	Month4	Month5
Lungs (g)	HbSS	0.9535	0.8858	0.0109	0.0030
	HbAS	0.9957	0.5742		
	HbAA	0.5533	0.6294	0.3170	0.0002
Lungs P-selectin (%)	HbSS				0.0457
	HbAA				0.2105
Lungs von Willebrand factor (%)	HbSS				0.0116
	HbAA				0.6471

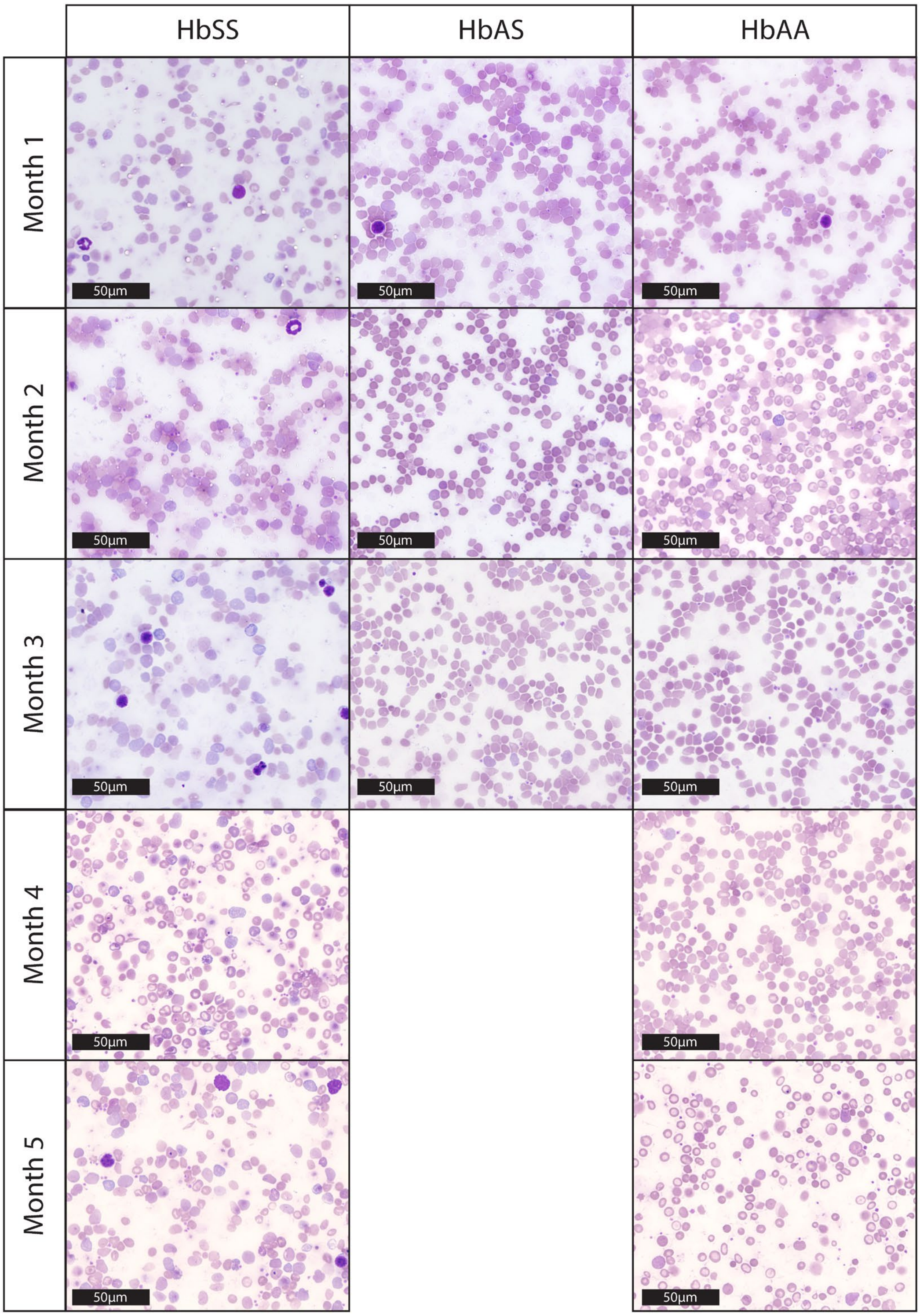

Fig. S5. Blood smears by age and genotype. Representative images of blood smears from HbSS, HbAS and HbAA mice at the studied timepoints (80x).

Biology Open • Supplementary information
